# Urban Onsite Sanitation Upgrades and Synanthropic Flies in Maputo, Mozambique: Effects on Enteric Pathogen Infection Risks

**DOI:** 10.1021/acs.est.2c06864

**Published:** 2022-12-14

**Authors:** Drew Capone, Zaida Adriano, Oliver Cumming, Seth R. Irish, Jackie Knee, Rassul Nala, Joe Brown

**Affiliations:** † Department of Environmental and Occupational Health, School of Public Health, Indiana University, 2719 E 10th St, Bloomington, Indiana47401, United States; ‡ WE Consult ltd, 177 Rua Tomas Ribeiro, Maputo1102, Mozambique; § Department of Disease Control, London School of Hygiene and Tropical Medicine, LondonWC1E 7HT, United Kingdom; ∥ Epidemiology and Public Health Department, Swiss Tropical and Public Health Institute, Kreuzstrasse 2, Allschwil4123, Switzerland; ⊥ Ministério da Saúde, Instituto Nacional de Saúde Maputo, Maputo1102, Mozambique; # Department of Environmental Sciences and Engineering, Gillings School of Public Health, University of North Carolina at Chapel Hill, Chapel Hill, North Carolina27599, United States

**Keywords:** flies, onsite sanitation, QMRA, enteric pathogens, infection, PCR

## Abstract

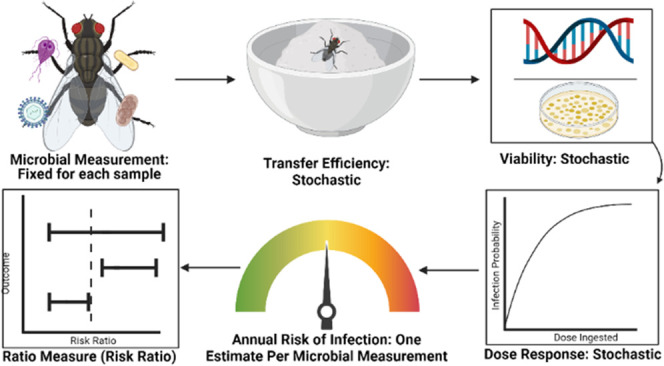

Synanthropic filth flies transport enteric pathogens from feces to food, which upon consumption poses an infection risk. We evaluated the effect of an onsite sanitation intervention—including fly control measures—in Maputo, Mozambique, on the risk of infection from consuming fly-contaminated food. After enumerating flies at intervention and control sites, we cultured fecal indicator bacteria, quantified gene copies for 22 enteric pathogens via reverse transcription quantitative polymerase chain reaction (RT-qPCR), and developed quantitative microbial risk assessment (QMRA) models to estimate annual risks of infection attributable to fly-contaminated foods. We found that the intervention reduced fly counts at latrine entrances by 69% (aRR = 0.31, [0.13, 0.75]) but not at food preparation areas (aRR = 0.92, [0.33, 2.6]). Half of (23/46) of individual flies were positive for culturable *Escherichia coli*, and we detected ≥1 pathogen gene from 45% (79/176) of flies, including enteropathogenic *E. coli* (37/176), adenovirus (25/176), *Giardia* spp. (13/176), and *Trichuris trichiura* (12/176). We detected ≥1 pathogen gene from half the flies caught in control (54%, 30/56) and intervention compounds (50%, 17/34) at baseline, which decreased 12 months post-intervention to 43% (23/53) at control compounds and 27% (9/33) for intervention compounds. These data indicate flies as a potentially important mechanical vector for enteric pathogen transmission in this setting. The intervention may have reduced the risk of fly-mediated enteric infection for some pathogens, but infrequent detection resulted in wide confidence intervals; we observed no apparent difference in infection risk between groups in a pooled estimate of all pathogens assessed (aRR = 0.84, [0.61, 1.2]). The infection risks posed by flies suggest that the design of sanitation systems and service delivery should include fly control measures to prevent enteric pathogen transmission.

## Abstract 2

Flies are implicated in enteric disease transmission in urban slums. Effective onsite sanitation may reduce infection risks from fly-contaminated foods.

## Introduction

The causal relationship between flies and disease has been hypothesized for centuries and understood since the germ theory of disease developed in the 1800s.^1−4^ Synanthropic filth flies—including houseflies (*Musca domestica*) and green bottle flies (*Lucilia sericata*)—feed on feces and can transport enteric pathogens in their alimentary canal and on their body.^1−4^ When a fly lands on food—or any other surface—it may vomit, defecate, or transfer enteric pathogens from its body onto the surface.^2,3^ These mechanisms enable flies to serve as vectors for pathogenic viruses, bacteria, protozoa, and helminths, which all may survive passage through the alimentary canal.^2,3^ Some bacteria may even proliferate in the fly gut^5,6^ and in fly regurgitate.^7^ The presence of enteric pathogens in food—via flies as a vector—then poses an infection risk to individuals upon consumption. This environmentally mediated transmission pathway is one of several enteric pathogen pathways,^8,9^ though flies remain understudied compared to other pathways such as drinking water.^10,11^

Some onsite sanitation technologies, such as ventilated improved pit latrines (VIPs) and pour-flush systems, use physical barriers that reduce opportunities for flies to breed. In a properly constructed VIP, the inside of the superstructure remains dark, and newly hatched flies will be attracted to the light in the ventilation pipe.^12^ A mesh screen at the top of the ventilation pipe prevents their escape; flies that cannot escape will die. Pour-flush systems may contain a water seal, which, if present, serves as a physical barrier between flies and the fecal material in the pit. Reduced access to breeding locations in the living environment may reduce fly counts and subsequently reduce the potential for flies to transport enteric pathogens from fecal wastes to the living environment.

Large controlled trials of water, sanitation, and hygiene^13−16^ found mixed effects of WASH interventions on children’s health outcomes. An intermediate outcome of some of these trials was to assess the impact of WASH interventions on enteric pathogens in the environment, and several studies measured the impact of these interventions on fecal indicator bacteria or pathogens in environmental matrices.^17−23^ Outcome variables to assess a WASH intervention’s impact have tended to include the binary presence of a target or its concentration in a specific environmental matrix. However, the risk of infection from environmental hazards is mediated by human behaviors, pathogen concentrations, and pathogen-specific dose–response relationships.^24^ Quantitative microbial risk assessment (QMRA)—a mechanistic framework for estimating health outcomes (e.g., infection and illness) using microbial measurements, exposure assessment, and pathogen-specific dose–response models—offers an alternative approach that considers how these additional factors influence transmission. Such an approach offers the potential for a more nuanced understanding of the interactions between WASH interventions, fecal contamination in the environment, and children’s health outcomes compared to epidemiological methods.

Community-scale insecticide application reduced childhood diarrhea by 24% in rural Gambia^25^ and 23% in Pakistan.^26^ A study of US military bases found intensive fly control via baited traps reduced clinic visits attributable to diarrhea by 42% and *Shigella* seroconversion by 76%.^27^ Unlike these communitywide interventions, onsite sanitation typically exists at the household level, and interventions may only target a subset of the population. In low-income informal settlements where onsite sanitation predominates, flies may be highly mobile between fecal wastes, food for human consumption, and other household surfaces. Flies can not only travel several kilometers in a single day but may also remain near common feeding locations, such as a pit latrine, for several days.^2^ It is then unclear if onsite sanitation interventions can reduce exposures to human food contaminated by flies. Our research aims were to (1) evaluate the enteric pathogen profile carried by flies in Maputo, Mozambique; (2) assess if a localized shared onsite sanitation reduced fly densities at latrine entrances and food preparation areas; and (3) estimate the impact of the intervention on a person’s annual risk of infection from consuming fly-contaminated food compared to a control group.

## Methods

### Study Setting

The Maputo Sanitation (MapSan) Trial was a controlled before-and-after trial that evaluated the effect of an urban onsite sanitation intervention on child health outcomes.^16^ The trial took place in low-income, informal neighborhoods in Maputo, Mozambique, where WASH conditions are poor and the burden of enteric disease is high.^16,17^ A nongovernmental organization delivered the intervention to compounds, which were occupied by multiple households that shared sanitation and a common outdoor living space. Sanitation facilities in poor condition at intervention compounds were replaced with shared pour-flush toilets that included septic tanks and soak-away pits. These intervention systems were built inside the compound boundary and were part of the households’ living environment. Neighborhood-level coverage was not the intention of the intervention; approximately 6% (*n* = 8601/145,000) of neighborhood residents received the intervention.^28^ The intervention infrastructure contained physical barriers—including mesh netting over ventilation pipes and water-seal toilets—that reduced the potential for flies to breed in the fecal sludge in the septic tank. Control compounds were concurrently enrolled from the same or adjacent neighborhoods as intervention compounds and continued using existing shared sanitation. Detailed descriptions of the inclusion criteria and the sanitation intervention are described elsewhere.^16,28^

### Sample Collection

We collected flies at latrine entrances and food preparation areas using sticky traps^29^ (Text S1) from a convenience sample of 50 control and 50 intervention compounds at baseline (pre-intervention) and 12 months following delivery of the intervention (median difference = 383 days, interquartile range = 372, 405). Enumerators hung individual rectangular blue sticky traps (pre-intervention: Suterra, Bend, Oregon; post-intervention: Great Lakes IPM, Vestaburg, Michigan) at least 1.5 m off the ground and within one meter of the latrine entrance and the food preparation area. Approximately 24 h later, the enumerator returned and recorded the number of flies on each trap. Then, the enumerator carefully removed each fly from the trap using tweezers that were sterilized with 10% bleach and 70% ethanol between compounds but not between flies. All flies caught in the traps were collected into Whirl-Pak bags (Nasco, Fort Atkinson, WI) pre-intervention and into sterile 15 mL centrifuge tubes (VWR, Radnor, PA) post-intervention. Flies were stored on ice and transported to laboratories at the Ministry of Health in Maputo, Mozambique. Samples were deposited into a freezer at −80 °C on the same day as collection. Some flies remained frozen at the Mozambican Ministry of Health for analysis, and the remainder were shipped from Maputo, Mozambique, to Atlanta, Georgia, on dry ice (−80 °C) with temperature monitoring for later molecular analysis.

### *Escherichia coli* Culture

We randomly selected 46 flies collected at baseline to measure the fecal indicator bacteria *E. coli* (Figure S1) following storage at −80 °C for approximately 4 years. We placed them into sterile tubes, determined the mass of each fly, crushed flies using a sterile disposable pestle (Kimble Chase, Vineland, NJ), added 3 mL of sterile phosphate buffered saline (Sigma-Aldrich, St. Louis MO), manually shook the tubes for 2 min, and then waited for 10 min for the solids to settle.^30^ Next, we pipetted 1 mL of the supernatant onto *E. coli*-specific Compact Dry plates (Compact Dry EC, VWR, Vienna, Austria), then incubated the plates at 37 °C for 24 h, and counted colony-forming units (CFUs). The remaining supernatant was stored at 4 °C, and if a plate was too numerous to count, the following day, the supernatant was diluted 1:100 and re-analyzed. Based on the manufacturer’s instructions and the dilutions used, the lower limit of detection (LOD) was 3 CFU *E. coli* per fly, and the upper limit of detection was approximately 10^4^ CFU *E. coli* per fly. We included one negative process control each day.

### Nucleic Acid Extraction

We aimed to extract nucleic acids from one fly per compound, for which we had flies available in Atlanta, Georgia. Given the heterogeneity in fly capture, we followed a procedure to select flies for analysis. From compounds where we caught a single fly, we analyzed the fly regardless of the species or location caught. In cases where we caught at least one housefly and bottle fly, we randomly selected a housefly because houseflies were caught more frequently than bottle flies. If we caught flies from both the latrine entrance and the food preparation area, we selected a fly from the food preparation area because these flies were more likely to land on food. Descriptive examples of this selection process are described in Text S2.

We used a modification of the Qiagen DNeasy Blood and Tissue Kit (Qiagen, Hilden, Germany) to extract total nucleic acids from 188 individual flies. First, we bead-beat flies for four cycles of 45 s in bead-beating tubes containing three sizes of glass beads and 180 μL Qiagen Buffer ATL. Next, we incubated the flies following the addition of another 180 μL of Buffer ATL, 40 μL of proteinase K (Qiagen, Hilden, Germany), and 6 μL of carrier RNA (Qiagen, Hilden, Germany) for 3 h at 56 °C. Then, we proceeded with extraction, following the manufacturer’s protocol. We spiked in approximately 10^7^ gene copies of bacteriophage MS2 (ATCC, Manassas, VA), an RNA phage, and 10^6^ copies of a synthetic DNA sequence (IDT, Coralville, IA) as our extraction-positive controls.^31^ On each day of extraction, we included at least one negative extraction control.

### TaqMan Array Card

We assayed nucleic acids using a custom TaqMan Array Card (TAC) (Thermo Fisher Scientific, Waltham, MA) that tested for 29 gene targets corresponding to 22 pathogens, following Liu et al.^32,33^ (Table S1), including nine bacteria (*Campylobacter jejuni/coli*, *Clostridium difficile*, Enteroaggregative *E. coli* (EAEC), *Shigella*/Enteroinvasive *E. coli* (EIEC), Enteropathogenic *E. coli* (EPEC), Enterotoxigenic *E. coli* (ETEC), Shiga toxin-producing *E. coli* (STEC), *Salmonella* spp., *Vibrio cholerae*), six viruses (adenovirus, astrovirus, pan-enterovirus, norovirus, rotavirus, sapovirus), three protozoa (*Cryptosporidium parvum* and *Cryptosporidium hominis*, *Entamoeba histolytica*, and *Giardia* spp.), and four helminths (*Ancylostoma duodenale*, *Ascaris lumbricoides*, *Necator americanus*, and *Trichuris trichiura*). The TAC also included targets for antimicrobial resistance genes; these data will be published separately. We combined 40 μL of template with 60 μL of AgPath-ID One-Step RT-PCR Master Mix (Thermo Fisher Scientific, Waltham, MA) and then added the mixture into each TAC port. Cards were centrifuged twice for 1 min at 1200 rpm, sealed, and trimmed. We performed reverse transcription quantitative PCR (RT-qPCR) using a QuantStudio 7 Flex instrument (Thermo Fisher Scientific, Waltham, MA) with the following thermocycling conditions: 45 °C for 20 min and 95 °C for 10 min, followed by 45 cycles of 95 °C for 30 s and 60 °C for 1 min. We manually set the threshold by comparing exponential curves and multicomponent plots with the positive control plots (Figure S2).^34,35^ Only amplification before a quantification cycle (Cq) of 40 was called as positive for a target, which was based off the performance of our negative controls (Tables S1 and S2).^17,36^

We developed a positive control, which was a plasmid that contained all target sequences, according to Kodani and Winchell, 2012.^37^ From an 8-fold serial dilution of this positive control, we ran standard curves to validate the performance of each assay and estimated gene copy concentrations. In addition, we ran dilutions ranging from 10^–1^ to 10^3^ gene copies per reaction well to determine the 95% limit of detection for each assay according to Stokdyk et al.^38^ On each day of TAC analysis, we included at least one negative extraction control and one PCR positive control.

### Digital PCR

We assayed extracted nucleic acids using digital PCR (dPCR) to test for the *E. coli*-specific gene, *ybbW*,^39^ with a QIAcuity Four instrument (Qiagen, Hilden, Germany). We combined 2 μL of template with 13.3 μL of QIAcuity EvaGreen PCR Master Mix (Qiagen, Hilden, Germany), forward and reverse primers (0.4 μM concentration), and DNase/RNase free water. After assay mixtures were pipetted into the wells of a QIAcuity 26k nanoplate, we sealed the plate and used the following thermocycling condition: 2 min at 95 °C, followed by 40 cycles of 95 °C for 15 s, 59 °C for 15 s, 72 °C for 15 s, and then a 5 min cool down at 40 °C. We included at least one PCR positive control and one PCR negative control on each plate. We manually set the threshold between distinct positive and negative bands.

### Exposure Assessment

We used the methods described in Capone et al.^40^ for each pathogen detected in at least 5% of intervention and control flies at the baseline phase and at least 5% in either study arm at the 12-month phase. These pathogens included *Giardia*, EPEC, EAEC, ETEC, adenovirus, and *T. trichiura*. For pathogens with multiple targets, we selected the largest gene copy value detected (Table S1).

The intervention was not associated with a difference in fly counts in the food preparation area, and we lacked site-specific data regarding fly contact with food. Consequently, we used QMRA to model a scenario where a single fly landed on food once a week immediately before consumption. This is a conservative estimate based on Lindeberg et al., which reported 1.1 fly landings per minute on uncovered rice in urban Dhaka, Bangladesh.^41^ The potential difference in QMRA-estimated infection risk between study arms is subsequently driven by the enteric pathogen concentration in flies and not fly counts.

We used the *fitdistrplus*(42) package in R to fit a log-normal distribution to the mass (mg) of individual flies we assessed. In our model, we divide the value reported by De Jesús et al.,^58^—that a fly transfers 0.1 mg of mass per landing on average—by a random value from this fly mass distribution. We used this method as a conservative estimate of the transfer efficiency of pathogens from a fly to food (median efficiency = 1.4%, interquartile range = 0.78, 2.6%). This estimate includes the total pathogens transferred from fly vomit, defecation, and mechanical transport from the fly body (Table S3).

### Dose Harmonization

Some of the pathogens we assessed contain multiple copies of the target sequence. We included point estimates and uniform distributions across the range of possible gene copies per genome based on values reported in the literature (Table S3).

### Infectious Unit

PCR assays measure nucleic acids from viable and nonviable organisms. We estimated pathogen viability using a ratio of colony-forming units (CFUs) of *E. coli* to gene copies of the *E. coli* gene *ybbW* for bacterial, viral, and protozoan pathogens. These measures were determined on separate flies, preventing direct comparison of matched values. Instead, we subtracted the median log_10_ transformed gene copies of *ybbW* per fly from the median log_10_ transformed CFUs per fly to generate a point estimate of viability. We used the average of the log_10_ transformed standard deviations from these two measures to represent the standard deviation around the viability point estimate. We input this log_10_ transformed point estimate and standard deviation as a normal distribution to propagate the variability of pathogen viability into our model. In addition, helminth ova are more persistent than other pathogens, so we assumed that 75% of *T. trichiura* ova were viable based on Steinbaum et al.,^18,43^ (Table S3).

### Dose–Response

We estimated the daily probability of infection for each pathogen using dose–response parameters taken from the literature (Table S3).

### Risk Characterization

**Figure 1:**
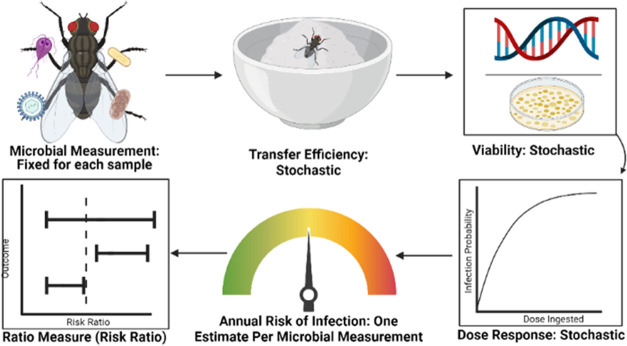
QMRA model overview.

We programmed the model as a Monte Carlo simulation in R (Figure 1). In the model, we fixed the microbial measurements to link the variance in our empirical data with our model outcome. Then, we randomly sampled from stochastic distributions to calculate a daily infection risk for each independent trial. We repeated the model 52 times, representing one exposure event per week for a year to calculate the annual risk of infection using eq 1.^44,45^ Then, to ensure the convergence of our estimates, we repeated this process 100 times (i.e., estimating 5200 weekly risks of infection and 100 annual risks of infection per microbial measurement) and used the median annual risk of infection for each microbial measurement from these 100 datasets as the outcome variable during hypothesis testing.
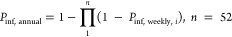
1

### Difference-in-Difference (DID) Analysis

The output of the risk characterization step was 176 annual estimates of infection risk—between 0 and 100%—directly tied to our microbial measurements, which included control and intervention compounds at baseline and the 12-month follow-up. We used a difference-in-difference (DID) approach^46^ to assess the impact of the intervention on fly counts at latrine entrances and food preparation areas and the estimated annual infection risk for each pathogen. In addition, we used to same methods to estimate the impact of the intervention on the pooled infection risk for a hypothetical pathogen by analyzing the risk estimates from the six most frequently detected pathogens in a single model. The DID approach is a quasi-experimental approach that typically uses longitudinal data from control and intervention groups. This approach relies on the parallel trend assumption, meaning that the initial difference between the two groups is assumed to remain constant over time. We used generalized estimating equations (GEE)^47^ to fit unadjusted Poisson regression models with robust standard errors and accounted for clustering at the compound level. As our infection risk estimates were linked to the original microbial measurements, we were also able to fit adjusted Poisson regression models that included the following covariates chosen *a priori*: fly species (bottle vs housefly), location the fly was caught (food preparation vs latrine), and wealth index.^48^ Adjusted models using fly count data as the outcome variable only included wealth index as a covariate.

### Power Analysis

We did not perform power analysis *a priori*, as the sample size was limited by fly capture and subsequent fly availability for nucleic acid extraction. Instead, we performed a post hoc power analysis using the WebPower package in R.^49^ While infrequent detection resulted in low power for individual pathogen estimates (Table S4), we had 80% power (α = 0.05) to observe a 23% reduction (RR = 0.77) in estimated infection risk for the pooled analysis.

### Sensitivity Analysis

Recognizing that infection risk estimates are dependent on the model parameters used, we re-ran alternative model scenarios to demonstrate how changes in model parameters impacted our results (Table S5). The parameters included a lower imputed value for nondetects (i.e., using the theoretical limit of detection), an alternative estimate for fly-food transfer efficiency based on a back-of-the-envelope calculation,^50−52^ viability estimates taken from the literature instead of our empirical data,^53,54^ and a scenario where flies land on food twice per week instead of once.

## Results

### Controls

From the 188 flies we analyzed on TAC, we excluded 12 because either the RNA or DNA extraction control did not amplify as expected. Our eight PCR positive controls (i.e., plasmids containing all target sequences) amplified as expected on each day we ran TAC. We did not observe amplification for any target before a C_q_ of 40 among our 12 negative extraction controls. The two negative process controls used to monitor our *E. coli* culture methods were also negative.

### Fly Prevalence and Counts

At baseline—combined from latrine entrances and food preparation areas—we caught a mean of 18 flies per intervention compound (95% CI: 13, 24) and 13 flies per control compound (95% CI: 9.6, 17). At the 12-month follow-up period, we caught fewer flies; the mean number of flies caught at intervention compounds was 3.2 (95% CI: 1.8, 4.7) and was 4.5 at control compounds (95% CI: 2.8, 6.2). Disaggregated between compound locations, the intervention reduced mean fly counts at latrine entrances by 69% (aRR = 0.31, [0.13, 0.75]) but had no effect on fly counts at food preparation areas (aRR = 0.92, [0.33, 2.6]). Fly counts and prevalence divided by phase, arm, and compound location can be found in Table S6.

### *E. coli*

**Figure 2:**
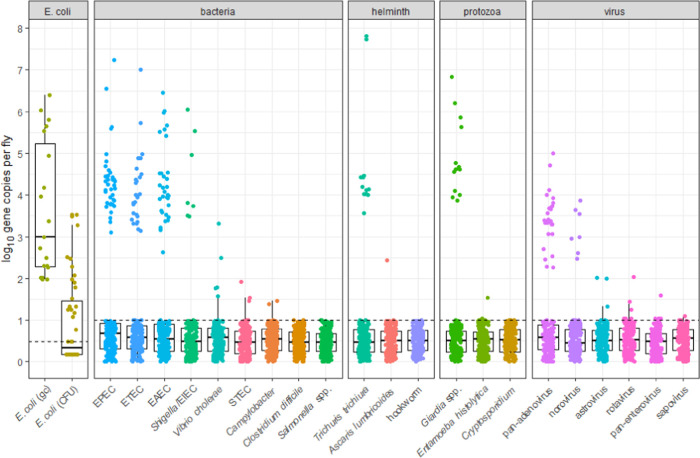
Culture and PCR results for all microbial targets. Note: All points below the dotted line were nondetects. We analyzed 19 flies for *E. coli* gene copies, 46 flies for culturable *E. coli*, and 176 flies for pathogen genes.

We found that half (23/46) of the flies analyzed were positive for culturable *E. coli*. The median concentration was 0.45 log_10_ CFU *E. coli* per fly, and the mean was 1.0 log_10_ (Figure 2). All extracted nucleic acids measured via dPCR (19/19) were positive for the *E.*coli-specific gene *ybbW*, with a median concentration of 3.0 log_10_ gene copies per fly and a mean of 3.6 log_10_ gene copies per fly (Figure 2).

### Pathogen Genes

We detected ≥1 pathogen gene from half the flies caught in control (54%, 30/56) and intervention compounds (50%, 17/34) at baseline. The prevalence decreased during the 12-month phase to 43% (23/53) for flies from control compounds and 27% (9/33) for flies from intervention compounds (Table 1). In addition, we detected a gene associated with each pathogen assayed using the TAC from ≥1 fly except for *C. difficile*, *Salmonella*, the two hookworm targets *A. duodenale* and *N. americanus*, and the two *Cryptosporidium* targets (Figure 2 and Table 1). The most prevalent bacterial pathogen was EPEC (37/176), helminth was *T. trichiura* (12/176), protozoa was *Giardia* (13/176), and virus was adenovirus (25/176) (Figure 2 and Table 1).

**Table 1:** Pathogens Detected in Flies and Estimated Infection Risks from Fly-Contaminated Food Consumption^a^

		pathogen prevalence in flies		mean annual risk of infection (minimum, maximum)		RR (95% CI)*	aRR (95% CI)
pathogen	trial Arm	baseline	12-month	baseline	12-month		
pooled infection risk (hypothetical pathogen)	control	NA†		NA†		0.83 (0.63, 1.1)	0.84 (0.61, 1.2)
	intervention						
enteropathogenic *E. coli*	control	25% (14/56)	15% (8/53)	20% (5.1%, >99%)	16% (6.0%, >99%)	1.1 (0.48, 2.7)	1.4 (0.57, 3.3)
	intervention	24% (8/34)	21% (7/33)	22% (4.9%, 94%)	19% (6.2%, >99%)		
adenovirus	control	21% (12/56)	7.6% (4/53)	20% (6.4%, 96%)	16% (9.1%, >99%)	0.60 (0.33, 1.1)	0.57 (0.30, 1.1)
	intervention	27% (9/34)	0% (0/33)	24% (4.8% >99%)	11% (9.5%, 13%)		
enteroaggregative *E. coli*	control	18% (10/56)	17% (9/53)	26% (4.3%, >99%)	26% (14%, >99%)	0.76 (0.49, 1.2)	0.73 (0.48, 1.1)
	intervention	27% (9/34)	15% (5/33)	34% (17%, >99%)	25% (17%, 85%)		
enterotoxigenic *E. coli*	control	16% (9/56)	13% (7/53)	29% (21%, >99%)	27% (20%, 91%)	0.92 (0.72, 1.2)	0.93 (0.74, 1.2)
	intervention	24% (8/34)	9.1% (3/33)	30% (19%, 73%)	26% (21%, 56%)		
*T. trichiura*	control	13% (7/56)	5.7% (3/53)	5.2% (1.3%, >99%)	2.3% (1.3%, 19%)	0.66 (0.19, 2.3)	0.46 (0.16, 1.3)
	intervention	5.9% (2/34)	0% (0/33)	5.3% (1.4%, >99%)	1.5% (1.4%, 1.7%)		
*Giardia*	control	7.1% (4/56)	7.6% (4/53)	13% (5.4%, >99%)	12% (5.3%, >99%)	**0.41 (0.18, 0.96)**	0.50 (0.23, 1.1)
	intervention	15% (5/34)	0% (0/33)	19% (5.7%, >99%)	6.5% (5.4%, 8.0%)		
*Shigella*/EIEC	control	5.4% (3/56)	3.8% (2/53)	NA‡			
	intervention	5.9% (2/34)	0% (0/33)				
norovirus GI/GII	control	5.4% (3/56)	3.8% (2/53)	NA‡			
	intervention	5.9% (2/34)	0 (0/53)				
rotavirus A	control	5.4% (3/56)	1.9% (1/53)	NA‡			
	intervention	5.9% (2/34)	0% (0/33)				
enterovirus	control	5.4% (3/56)	0% (0/53)	NA‡			
	intervention	5.9% (2/34)	0% (0/33)				
*V. cholerae*	control	3.6% (2/56)	0% (0/53)	NA‡			
	intervention	2.9% (1/34)	6.1% (2/33)				
astrovirus	control	1.8% (1/56)	0% (0/53)	NA‡			
	intervention	5.9% (2/34)	0% (0/33)				
*C. jejuni/coli*	control	1.8% (1/56)	1.9% (1/53)	NA‡			
	intervention	0% (0/34)	0% (0/33)				
shiga-toxin producing *E. coli*	control	0% (0/56)	1.9% (1/53)	NA‡			
	intervention	2.9% (1/34)	3.0% (1/33)				
*A. lumbricoides*	control	0% (0/56)	0% (0/53)	NA‡			
	intervention	2.9% (1/34)	0% (0/33)				
sapovirus	control	0% (0/56)	0% (0/53)	NA‡			
	intervention	2.9% (1/34)	0% (0/33)				
*E. histolytica*	control	0% (0/34)	3.8% (2/53)	NA‡			
	intervention	0% (0/56)	0% (0/33)				
≥1 pathogen gene detected	control	54% (30/56)	43% (23/53)				
	intervention	50% (17/34)	27% (9/33)				

a Note: *Indicates the risk of enteric pathogen infection in the intervention group compared to the control group. †Represents the pooled infection risk of enteropathogenic *E. coli*, adenovirus, enteroaggregative *E. coli*, enterotoxigenic *E. coli*, *T. trichiura*, and *Giardia*; there is no corresponding prevalence or annual infection risk for this estimate. Bold indicates *p* < 0.05. The following pathogens were not detected: *A. duodenale*, *C. difficile*, *C. parvum*, *C. hominis*, *N. americanus*, and *Salmonella*.

### Annual Risk of Infection

As we did not detect any pathogen in more than 50% of flies, the median annual risk of infection for each pathogen was calculated from an imputed value for both study arms and phases, resulting in homogeneous median risk estimates. The annual risk estimates approached 100% for most data above the limit of detection (Table 1).

### Intervention Impact

According to the traditional definition of statistical significance,^55^ we estimated a significant reduction in the annual risk of infection from *Giardia* (RR = 0.41, [0.18, 0.96]) in the unadjusted model (Table 1). Likewise, the adjusted point estimates for five of the six most frequently detected pathogens—adenovirus (aRR = 0.57, [0.30, 1.1]), EAEC (aRR = 0.73, [aRR = 0.48, 1.1]), ETEC (aRR = 0.93, [0.74, 1.2]), *T. trichiura* (aRR = 0.46, [0.16, 1.3]), and *Giardia* (aRR = 0.50, [0.23, 1.1)]—also suggested the intervention had a protective effect. In addition, the point estimate for pooled infection risk indicated a protective effect (aRR = 0.84, [0.61, 1.2]). However, the confidence intervals included one for these five pathogens and the pooled risk estimate, indicating that the intervention may have had no effect or even increased the risk of infection compared to the control group.

### Sensitivity Analysis

Changes in model parameters did not affect the directionality of point estimates but did result in changes to individual point estimates (Table S7). Setting nondetects to the theoretical limit of detection (LOD)—instead of imputing them from zero to 95% LOD—resulted in lower infection risk estimates for nondetect samples. Models using this lower LOD suggested the intervention reduced the fly-mediated risk of infection for adenovirus (aRR = 0.32, [0.14, 0.97]), *Trichuris* (aRR = 0.12, [0.03, 0.48]), and *Giardia* (aRR = 0.19, [0.05, 0.80]) but not for the pooled infection risk estimate (aRR = 0.75, [0.42, 1.4]). Using viability estimates from the literature for *Giardia* and adenovirus—instead of our empirical data—resulted in nearly the same result for *Giardia* (aRR = 0.46, [0.20, 1.1]) but suggested a protective effect for adenovirus (aRR = 0.08, [0.02, 0.42]). An alternative estimate of transfer efficiency (3.2%)—instead of our stochastic method—also resulted in similar infection risk estimates. Except, this model suggested the intervention reduced the fly-mediated risk of infection by adenovirus (aRR = 0.73, [0.52, 1.0]) and *Trichuris* (aRR = 0.14, [0.04, 0.53]). Finally, doubling the frequency of fly contact with food also resulted in similar infection risk estimates (pooled aRR = 0.84, [0.58, 1.2]).

## Discussion

A large body of literature from the 1880s^56^ to the mid-1900s^1−4^ demonstrated that flies were capable of transporting enteric pathogens on body surfaces and in the alimentary canal. While these early studies were limited by the methods available, application of the methods available in the 21st Century has broadened our understanding of the link between flies and disease. Some of these studies have focused on culturing fecal indicator bacteria from flies,^21,57^ the potential contamination of food,^7,41,58^ fly densities,^59^ and fly-mediated spread of antimicrobial resistance.^60,61^ According to a 2018 systematic review of human pathogens carried by flies,^62^ our novel multipathogen assessment of individual flies represents the first PCR-based detection of human enteric viruses (i.e., adenovirus, norovirus, astrovirus, enterovirus, and sapovirus) from wild caught flies. The high prevalence and concentration of pathogens we detected in flies, combined with their mobility in the living environment, suggests flies may be capable of distributing any enteric pathogen present in fecal wastes to surfaces and food where the opportunity for such transmission exists.

We infrequently detected individual pathogens, which resulted in low power for these regression model estimates and wide confidence intervals. While low power suggests a cautious interpretation of individual pathogen estimates, there was a general trend; five of six individual point estimates showed the onsite sanitation intervention may have reduced the fly-mediated risk of enteric infection.^55^ In addition, the general trend was reproduced in the sensitivity analyses. However, the results clearly indicated that the shared urban onsite sanitation intervention did not dramatically reduce the QMRA-estimated infection risk from fly-contaminated foods after 1 year, and wide confidence intervals indicated the intervention may have increased the risk of infection. These results corroborate other studies of environmental fecal contamination as part of the MapSan trial during the 12-^63^ and 24-month^17^ phases, which found the intervention may have had a small protective effect against some enteric pathogens in latrine entrance soils, but levels of fecal contamination remained high post-intervention. While the intervention was associated with a reduction in fly counts at latrine entrances, we observed no difference at food preparation areas, which is where fly contact with food is more likely to occur.

Two community wide fly control interventions—that used insecticide treatment^25^ and baited traps^27^—reduced fly counts by approximately 75%. A study in Pakistan found that village-scale insecticide spraying reduced fly counts by more than 90%, though baited traps had no effect.^26^ At both control and intervention compounds, there was a substantial decrease in fly counts from baseline to the 12-month phase. However, this finding may have been the result of the methods we used. The manufacturer discontinued the fly trap used at baseline, and anecdotally, field workers reported the fly traps used post-intervention were not as sticky as those used pre-intervention. In addition, fly traps capture a subset of the flies from an area of interest as they must compete with alternative sites (e.g., feces, fecal sludge, and solid waste).^29^ The actual number of flies inside study compounds was likely greater than indicated by our fly count results.

The high prevalence and concentration of culturable *E. coli* and pathogen-associated genes we detected suggest that the fly-food-ingestion pathway may be underappreciated in Maputo and similar informal low-income communities. Effective fly control strategies are essential to reduce risks. These strategies can be classified as either source or contact control. Certain onsite sanitation systems that are properly constructed and maintained—such as VIPs and pour-flush systems—offer the opportunity for source control by preventing fly breeding,^12^ whereas contact control strategies include fly traps, insecticides, and covering food.^26,41^ Contact control strategies may be easier to implement in the near future compared to investments in sanitation infrastructure. However,^64,65^ there is mixed evidence that fly trap interventions reduce fly counts^26,27^ and some insects—including flies—are evolving resistance to insecticides.^64,65^

Physical barriers to limit fly breeding—namely mesh screens over ventilation pipes and water seals—would not require the same degree of ongoing effort as contract control strategies. However, this infrastructure will deteriorate after a few years without consistent maintenance^12^ and may need to achieve a certain threshold of community coverage to reduce fly counts at locations relevant to exposure (i.e., food preparation areas). The sanitation intervention we evaluated included a water seal and mesh screen over a ventilation pipe. These physical barriers may explain the large reduction in fly counts at intervention latrine entrances compared to controls. In addition to human excreta, efforts to control fly breeding in low- and middle-income countries are complicated by the presence of animal feces, which may exceed the quantity of human feces in some settings.^66^ The difficulty associated with managing animal waste suggests alternative and sustainable fly control strategies may be needed to dramatically reduce the infection risks posed by fly-contaminated food in Maputo and similar informal urban settlements.

A wide range of media is suitable for fly larval development. Houseflies have been observed to reproduce in human and animal excrement, fecal sludges, decaying foods, household refuse, and solid wastes.^3^ The high water content of septic tank sludge may prevent flies from breeding. However, Hargreaves observed large numbers of flies breeding in the scum layer of septic tanks.^67^ We did not attempt to observe fly larvae in latrines and septic tanks, and it is unclear what role—if any—sludge characteristics played in fly reproduction habits.

Flies may be able to transmit enteric pathogens from feces to food: this pathway is directly observable and has been used widely in Community-Led Total Sanitation (CLTS) programming to exemplify exposure risks of uncontained fecal contamination.^68^ Well-fed houseflies have been observed to defecate approximately every four and a half minutes.^3^ Although pathogen residence time in the alimentary canal varies, Sieyro, 1942,^69^ found cysts of *E. histolytica* could be passed by houseflies in as little as 1 min and were detectable up to 34 h after feeding. In addition, flies often regurgitate their food, but the structure of the proboscis selectively filters some protozoan cysts and helminth ova such that they are more likely to be shed in feces than vomit.^2^ After vomiting, flies may re-ingest this material to aid with mechanical digestion.^2^ Graham-Smith,^50^ and Wenyon and O’Connor,^51^ reported that houseflies fed once on milk produced 16–31 specks (i.e., vomitus or excreta) per fly, most of which they classified as vomit. Houseflies cannot typically swallow particles larger the 40 μm in diameter, which may prevent them from ingesting some *Ascaris* ova, but oblong *Trichuris* ova could be swallowed lengthwise (minimum diameter ∼ 20–25 μm). This size limitation may be why we detected *Trichuris* more frequently than *Ascaris* (minimum diameter ∼45 μm) despite *Ascaris* being more prevalent in fecal sludges collected from trial compounds (Table S8).^70^ In addition to the abundance of vomit and excreta that flies generate, they have a compulsion for cleaning. Flies constantly preen their wings or brush their body parts, which dislodges organisms that may have become attached to their integument.^2^

There are several limitations to consider as part of our study. First, we sterilized tweezers between compounds and not individual flies, which may have led to contamination between flies from the same compound. Second, we lacked empirical evidence to describe the frequency of fly contact with food, though we used a highly conservative estimate based on Lindeberg et al.^41^ Further, we conservatively estimated equal contact between flies and food among study arms. While we observed no difference in fly counts at food preparation areas, the substantial reduction in fly counts at latrine entrances suggests we may have underestimated the impact of the intervention. Third, there is a paucity of literature quantifying the transfer of pathogens from flies to food, but we used a conservative estimate based on values reported in the literature. Fourth, the applicability of dose–response relationships developed in high-income countries to individuals living in informal settlements is unclear. Endemic exposure may result in acquired immunity, but repeated enteric infections may also compromise the immune system, leading to greater susceptibility.^71^ Further, the prevalence of individual pathogens was low because we extracted nucleic acids from individual flies—rather than pools of flies—and the LOD for TAC is higher than traditional qPCR or dPCR.^72^ Preamplification of nucleic acid extracts is an alternative method to lower the high LOD associated with the TAC workflow.^34^ In addition, low pathogen prevalence likely resulted in limited power to observe significant effects. However, our results indicate that the intervention did not result in substantial reductions in QMRA-estimated infection risks, which is similar to the null effect observed in the main outcome of the MapSan trial.^16^

Viability is a key variable in QMRA;^73^ we used an empirical ratio of culturable to gene copies of *E. coli*. This approach may have underestimated infection risks from *Giardia* and adenovirus, as these pathogens may be more persistent than *E. coli*,^74,75^ but we offered an alternative viability estimate for these pathogens in the sensitivity analysis. However, this alternative used an estimate that likely relied on an assumption of unknown and unknowable validity. And the feeding habits of houseflies and green bottle flies in this setting—namely on fresh feces—gives credence to our estimates that some pathogen-specific genes came from viable organisms. Additionally, flies were frozen before culturing, which may have resulted in an underestimate of viability and subsequently resulted in an underestimate of annual infection risks.

Flies are capable of transporting enteric pathogens from feces to food and other surfaces, which may then cause enteric infection and illness. We used QMRA to demonstrate that the fly-food pathway is highly plausible and may contribute to the burden of enteric disease in low-income Maputo and similar settings.^26^ We found some evidence to suggest that the onsite sanitation intervention reduced the annual risk of infection from consuming fly-contaminated food, but low power limited the interpretation of effects for individual pathogens. The onsite sanitation intervention we assessed was not implemented at a community level, indicating it is possible the intervention did not reach an adequate threshold of community coverage to substantially reduce infection risks from fly-contaminated foods. In addition, the intervention fly barriers—mesh netting over the ventilation pipe, the water seal, and the sealed septic tank—may have become damaged post-intervention and enabled flies to breed in the fecal sludge. Transformative WASH^30,76^ interventions should consider the maintenance of fly barriers and fly contact control interventions (e.g., traps, insecticides, and covering food) as necessary components of holistic interventions that might achieve substantial reductions in environmental fecal contamination and improve children’s health outcomes.

## Supporting Information Available

Fly trap piloting (Text S1); plots of *E. coli* concentrations (Figure S1); fly selection process (Text S2); TAC performance (Table S1); RT-qPCR plots (Figure S2); MIQE checklist (Table S2); QMRA parameters (Table S3); post hoc power (Table S4); sensitivity analysis parameters (Table S5); fly counts and prevalence (Table S6); sensitivity analysis results (Table S7); and pathogen prevalence comparison (Table S8) (PDF)

## References

[ref1] Hewitt C. G. House-Flies and How They Spread Disease, 1st ed.; Cambridge University Press: London, UK, 1912.

[ref2] Greenberg B. Flies and Disease Volume II: Biology and Disease Transmission, 1st ed.; Princeton University Press: Princeton, NJ, 1973.

[ref3] West L. S. The Housefly: Its Natural History, Medical Importance, and Control, 1st ed.; Comstock Publishing Company: Binghamton, NY, 1951.

[ref4] Howard L. O. The House Fly Disease Carrier: An Accounts of Its Dangerous Activities and of the Means of Destroying It, 1st ed.; Stokes Company: New York, 1911.

[ref5] Agui N.; Kobayashi M.; Sasaki T.; Saito N.; Tamura K.; Suzuki K.; Watanabe H. Houseflies: Not Simple Mechanical Vectors of Enterohemorrhagic *Escherichia coli* O157:H7. Am. J. Trop. Med. Hyg. 1999, 61, 625–629. 10.4269/ajtmh.1999.61.625. 10548298

[ref6] Greenberg B.; Kowalski J. A.; Klowden M. J. Factors Affecting the Transmission of Salmonella by Flies: Natural Resistance to Colonization and Bacterial Interference. Infect. Immun. 1970, 2, 800–809. 10.1128/iai.2.6.800-809.1970. 16557919 PMC416094

[ref7] Wasala L.; Talley J. L.; DeSilva U.; Fletcher J.; Wayadande A. Transfer of *Escherichia coli* O157:H7 to Spinach by House Flies, *Musca domestica* (Diptera: Muscidae). Phytopathology 2013, 103, 373–380. 10.1094/PHYTO-09-12-0217-FI. 23425236

[ref8] Wagner E.; Lanoix J. Excreta Disposal for Rural Areas and Small Communities. Monogr. Ser. World Health Organ. 1958, 39, 1–182. 13581743

[ref9] Penakalapati G.; Swarthout J.; Delahoy M. J.; McAliley L.; Wodnik B.; Levy K.; Freeman M. C. Exposure to Animal Feces and Human Health: A Systematic Review and Proposed Research Priorities. Environ. Sci. Technol. 2017, 51, 11537–11552. 10.1021/acs.est.7b02811. 28926696 PMC5647569

[ref10] Goddard F. G. B.; Ban R.; Barr D. B.; Brown J.; Cannon J.; Colford J. M.; Eisenberg J. N. S.; Ercumen A.; Petach H.; Freeman M. C.; Levy K.; Luby S. P.; Moe C.; Pickering A. J.; Sarnat J. A.; Stewart J.; Thomas E.; Taniuchi M.; Clasen T. Measuring Environmental Exposure to Enteric Pathogens in Low-Income Settings: Review and Recommendations of an Interdisciplinary Working Group. Environ. Sci. Technol. 2020, 11673–11691. 10.1021/acs.est.0c02421. 32813503 PMC7547864

[ref11] Sclar G. D.; Penakalapati G.; Amato H. K.; Garn J. V.; Alexander K.; Freeman M. C.; Boisson S.; Medlicott K. O.; Clasen T. Assessing the Impact of Sanitation on Indicators of Fecal Exposure along Principal Transmission Pathways: A Systematic Review. Int. J. Hyg. Environ. Health 2016, 219, 709–723. 10.1016/j.ijheh.2016.09.021. 27720133

[ref12] Mara D. D. The Design of Ventilated Improved Pit Latrines; International Bank for Reconstruction and Development/The World Bank, 1984.

[ref13] Luby S. P.; Rahman M.; Arnold B. F.; Unicomb L.; Ashraf S.; Winch P. J.; Stewart C. P.; Begum F.; Hussain F.; Benjamin-Chung J.; Leontsini E.; Naser A. M.; Parvez S. M.; Hubbard A. E.; Lin A.; Nizame F. A.; Jannat K.; Ercumen A.; Ram P. K.; Das K. K.; Abedin J.; Clasen T. F.; Dewey K. G.; Fernald L. C.; Null C.; Ahmed T.; Colford J. M. Effects of Water Quality, Sanitation, Handwashing, and Nutritional Interventions on Diarrhoea and Child Growth in Rural Bangladesh: A Cluster Randomised Controlled Trial. Lancet Glob. Health 2018, 6, e302–e315. 10.1016/S2214-109X(17)30490-4. 29396217 PMC5809718

[ref14] Null C.; Stewart C. P.; Pickering A. J.; Dentz H. N.; Arnold B. F.; Arnold C. D.; Benjamin-Chung J.; Clasen T.; Dewey K. G.; Fernald L. C. H.; Hubbard A. E.; Kariger P.; Lin A.; Luby S. P.; Mertens A.; Njenga S. M.; Nyambane G.; Ram P. K.; Colford J. M. Effects of Water Quality, Sanitation, Handwashing, and Nutritional Interventions on Diarrhoea and Child Growth in Rural Kenya: A Cluster-Randomised Controlled Trial. Lancet Glob. Health 2018, 6, e316–e329. 10.1016/S2214-109X(18)30005-6. 29396219 PMC5809717

[ref15] Humphrey J. H.; Mbuya M. N. N.; Ntozini R.; Moulton L. H.; Stoltzfus R. J.; Tavengwa N. V.; Mutasa K.; Majo F.; Mutasa B.; Mangwadu G.; Chasokela C. M.; Chigumira A.; Chasekwa B.; Smith L. E.; Tielsch J. M.; Jones A. D.; Manges A. R.; Maluccio J. A.; Prendergast A. J.; Humphrey J. H.; Jones A. D.; Manges A.; Mangwadu G.; Maluccio J. A.; Mbuya M. N. N.; Moulton L. H.; Ntozini R.; Prendergast A. J.; Stoltzfus R. J.; Tielsch J. M.; Chasokela C.; Chigumira A.; Heylar W.; Hwena P.; Kembo G.; Majo F. D.; Mutasa B.; Mutasa K.; Rambanepasi P.; Sauramba V.; Tavengwa N. V.; Van Der Keilen F.; Zambezi C.; Chidhanguro D.; Chigodora D.; Chipanga J. F.; Gerema G.; Magara T.; Mandava M.; Mavhudzi T.; Mazhanga C.; Muzaradope G.; Mwapaura M. T.; Phiri S.; Tengende A.; Banda C.; Chasekwa B.; Chidamba L.; Chidawanyika T.; Chikwindi E.; Chingaona L. K.; Chiorera C. K.; Dandadzi A.; Govha M.; Gumbo H.; Gwanzura K. T.; Kasaru S.; Makasi R.; Matsika A. M.; Maunze D.; Mazarura E.; Mpofu E.; Mushonga J.; Mushore T. E.; Muzira T.; Nembaware N.; Nkiwane S.; Nyamwino P.; Rukobo S. D.; Runodamoto T.; Seremwe S.; Simango P.; Tome J.; Tsenesa B.; Amadu U.; Bangira B.; Chiveza D.; Hove P.; Jombe H. A.; Kujenga D.; Madhuyu L.; Makoni P. M.; Maramba N.; Maregere B.; Marumani E.; Masakadze E.; Mazula P.; Munyanyi C.; Musanhu G.; Mushanawani R. C.; Mutsando S.; Nazare F.; Nyarambi M.; Nzuda W.; Sigauke T.; Solomon M.; Tavengwa T.; Biri F.; Chafanza M.; Chaitezvi C.; Chauke T.; Chidzomba C.; Dadirai T.; Fundira C.; Gambiza A. C.; Godzongere T.; Kuona M.; Mafuratidze T.; Mapurisa I.; Mashedze T.; Moyo N.; Musariri C.; Mushambadope M.; Mutsonziwa T. R.; Muzondo A.; Mwareka R.; Nyamupfukudza J.; Saidi B.; Sakuhwehwe T.; Sikalima G.; Tembe J.; Chekera T. E.; Chihombe O.; Chikombingo M.; Chirinda T.; Chivizhe A.; Hove R.; Kufa R.; Machikopa T. F.; Mandaza W.; Mandongwe L.; Manhiyo F.; Manyaga E.; Mapuranga P.; Matimba F. S.; Matonhodze P.; Mhuri S.; Mike J.; Ncube B.; Nderecha W. T. S.; Noah M.; Nyamadzawo C.; Penda J.; Saidi A.; Shonhayi S.; Simon C.; Tichagwa M.; Chamakono R.; Chauke A.; Gatsi A. F.; Hwena B.; Jawi H.; Kaisa B.; Kamutanho S.; Kaswa T.; Kayeruza P.; Lunga J.; Magogo N.; Manyeruke D.; Mazani P.; Mhuriyengwe F.; Mlambo F.; Moyo S.; Mpofu T.; Mugava M.; Mukungwa Y.; Muroyiwa F.; Mushonga E.; Nyekete S.; Rinashe T.; Sibanda K.; Chemhuru M.; Chikunya J.; Chikwavaire V. F.; Chikwiriro C.; Chimusoro A.; Chinyama J.; Gwinji G.; Hoko-Sibanda N.; Kandawasvika R.; Madzimure T.; Maponga B.; Mapuranga A.; Marembo J.; Matsunge L.; Maunga S.; Muchekeza M.; Muti M.; Nyamana M.; Azhuda E.; Bhoroma U.; Biriyadi A.; Chafota E.; Chakwizira A.; Chamhamiwa A.; Champion T.; Chazuza S.; Chikwira B.; Chingozho C.; Chitabwa A.; Dhurumba A.; Furidzirai A.; Gandanga A.; Gukuta C.; Macheche B.; Marihwi B.; Masike B.; Mutangandura E.; Mutodza B.; Mutsindikwa A.; Mwale A.; Ndhlovu R.; Nduna N.; Nyamandi C.; Ruvata E.; Sithole B.; Urayai R.; Vengesa B.; Zorounye M.; Bamule M.; Bande M.; Chahuruva K.; Chidumba L.; Chigove Z.; Chiguri K.; Chikuni S.; Chikwanda R.; Chimbi T.; Chingozho M.; Chinhamo O.; Chinokuramba R.; Chinyoka C.; Chipenzi X.; Chipute R.; Chiribhani G.; Chitsinga M.; Chiwanga C.; Chiza A.; Chombe F.; Denhere M.; Dhamba E.; Dhamba M.; Dube J.; Dzimbanhete F.; Dzingai G.; Fusira S.; Gonese M.; Gota J.; Gumure K.; Gwaidza P.; Gwangwava M.; Gwara W.; Gwauya M.; Gwiba M.; Hamauswa J.; Hlasera S.; Hlukani E.; Hotera J.; Jakwa L.; Jangara G.; Janyure M.; Jari C.; Juru D.; Kapuma T.; Konzai P.; Mabhodha M.; Maburutse S.; Macheka C.; Machigaya T.; Machingauta F.; Machokoto E.; Madhumba E.; Madziise L.; Madziva C.; Madzivire M.; Mafukise M.; Maganga M.; Maganga S.; Mageja E.; Mahanya M.; Mahaso E.; Mahleka S.; Makanhiwa P.; Makarudze M.; Makeche C.; Makopa N.; Makumbe R.; Mandire M.; Mandiyanike E.; Mangena E.; Mangiro F.; Mangwadu A.; Mangwengwe T.; Manhidza J.; Manhovo F.; Manono I.; Mapako S.; Mapfumo E.; Mapfumo T.; Mapuka J.; Masama D.; Masenge G.; Mashasha M.; Mashivire V.; Matunhu M.; Mavhoro P.; Mawuka G.; Mazango I.; Mazhata N.; Mazuva D.; Mazuva M.; Mbinda F.; Mborera J.; Mfiri U.; Mhandu F.; Mhike C.; Mhike T.; Mhuka A.; Midzi J.; Moyo S.; Mpundu M.; Msekiwa N.; Msindo D.; Mtisi C.; Muchemwa G.; Mujere N.; Mukaro E.; Muketiwa K.; Mungoi S.; Munzava E.; Muoki R.; Mupura H.; Murerwa E.; Murisi C.; Muroyiwa L.; Muruvi M.; Musemwa N.; Mushure C.; Mutero J.; Mutero P.; Mutumbu P.; Mutya C.; Muzanango L.; Muzembi M.; Muzungunye D.; Mwazha V.; Ncube T.; Ndava T.; Ndlovu N.; Nehowa P.; Ngara D.; Nguruve L.; Nhigo P.; Nkiwane S.; Nyanyai L.; Nzombe J.; Office E.; Paul B.; Pavari S.; Ranganai S.; Ratisai S.; Rugara M.; Rusere P.; Sakala J.; Sango P.; Shava S.; Shekede M.; Shizha C.; Sibanda T.; Tapambwa N.; Tembo J.; Tinago N.; Tinago V.; Toindepi T.; Tovigepi J.; Tuhwe M.; Tumbo K.; Zaranyika T.; Zaru T.; Zimidzi K.; Zindo M.; Zindonda M.; Zinhumwe N.; Zishiri L.; Ziyambi E.; Zvinowanda J.; Bepete E.; Chiwira C.; Chuma N.; Fari A.; Gavi S.; Gunha V.; Hakunandava F.; Huku C.; Hungwe G.; Maduke G.; Manyewe E.; Mapfumo T.; Marufu I.; Mashiri C.; Mazenge S.; Mbinda E.; Mhuri A.; Muguti C.; Munemo L.; Musindo L.; Ngada L.; Nyembe D.; Taruvinga R.; Tobaiwa E.; Banda S.; Chaipa J.; Chakaza P.; Chandigere M.; Changunduma A.; Chibi C.; Chidyagwai O.; Chidza E.; Chigatse N.; Chikoto L.; Chingware V.; Chinhamo J.; Chinhoro M.; Chiripamberi A.; Chitavati E.; Chitiga R.; Chivanga N.; Chivese T.; Chizema F.; Dera S.; Dhliwayo A.; Dhononga P.; Dimingo E.; Dziyani M.; Fambi T.; Gambagamba L.; Gandiyari S.; Gomo C.; Gore S.; Gundani J.; Gundani R.; Gwarima L.; Gwaringa C.; Gwenya S.; Hamilton R.; Hlabano A.; Hofisi E.; Hofisi F.; Hungwe S.; Hwacha S.; Hwara A.; Jogwe R.; Kanikani A.; Kuchicha L.; Kutsira M.; Kuziyamisa K.; Kuziyamisa M.; Kwangware B.; Lozani P.; Mabuto J.; Mabuto V.; Mabvurwa L.; Machacha R.; Machaya C.; Madembo R.; Madya S.; Madzingira S.; Mafa L.; Mafuta F.; Mafuta J.; Mahara A.; Mahonye S.; Maisva A.; Makara A.; Makover M.; Mambongo E.; Mambure M.; Mandizvidza E.; Mangena G.; Manjengwa E.; Manomano J.; Mapfumo M.; Mapfurire A.; Maphosa L.; Mapundo J.; Mare D.; Marecha F.; Marecha S.; Mashiri C.; Masiya M.; Masuku T.; Masvimbo P.; Matambo S.; Matarise G.; Matinanga L.; Matizanadzo J.; Maunganidze M.; Mawere B.; Mawire C.; Mazvanya Y.; Mbasera M.; Mbono M.; Mhakayakora C.; Mhlanga N.; Mhosva B.; Moyo N.; Moyo O.; Moyo R.; Mpakami C.; Mpedzisi R.; Mpofu E.; Mpofu E.; Mtetwa M.; Muchakachi J.; Mudadada T.; Mudzingwa K.; Mugwira M.; Mukarati T.; Munana A.; Munazo J.; Munyeki O.; Mupfeka P.; Murangandi G.; Muranganwa M.; Murenjekwa J.; Muringo N.; Mushaninga T.; Mutaja F.; Mutanha D.; Mutemeri P.; Mutero B.; Muteya E.; Muvembi S.; Muzenda T.; Mwenjota A.; Ncube S.; Ndabambi T.; Ndava N.; Ndlovu E.; Nene E.; Ngazimbi E.; Ngwalati A.; Nyama T.; Nzembe A.; Pabwaungana E.; Phiri S.; Pukuta R.; Rambanapasi M.; Rera T.; Samanga V.; Shirichena S.; Shoko C.; Shonhe M.; Shuro C.; Sibanda J.; Sibangani E.; Sibangani N.; Sibindi N.; Sitotombe M.; Siwawa P.; Tagwirei M.; Taruvinga P.; Tavagwisa A.; Tete E.; Tete Y.; Thandiwe E.; Tibugari A.; Timothy S.; Tongogara R.; Tshuma L.; Tsikira M.; Tumba C.; Watinaye R.; Zhiradzango E.; Zimunya E.; Zinengwa L.; Ziupfu M.; Ziyambe J.; Church J. A.; Desai A.; Fundira D.; Gough E.; Kambarami R. A.; Matare C. R.; Malaba T. R.; Mupfudze T.; Ngure F.; Smith L. E.; Curtis V.; Dickin K. L.; Habicht J.-P.; Masimirembwa C.; Morgan P.; Pelto G. H.; Sheffner-Rogers C.; Thelingwani R.; Turner P.; Zungu L.; Makadzange T.; Mujuru H. A.; Nyachowe C.; Chakadai R.; Chanyau G.; Makamure M. G.; Chiwariro H.; Mtetwa T.; Chikunya J.; Maguwu L.; Nyadundu S.; Moyo T.; Chayima B.; Mvindi L.; Rwenhamo P.; Muzvarwandoga S.; Chimukangara R.; Njovo H.; Makoni T. Independent and Combined Effects of Improved Water, Sanitation, and Hygiene, and Improved Complementary Feeding, on Child Stunting and Anaemia in Rural Zimbabwe: A Cluster-Randomised Trial. Lancet Glob. Health 2019, 7, e132–e147. 10.1016/S2214-109X(18)30374-7. 30554749 PMC6293965

[ref16] Knee J.; Sumner T.; Adriano Z.; Anderson C.; Bush F.; Capone D.; Casmo V.; Holcomb D. A.; Kolsky P.; MacDougall A.; Molotkova E.; Braga J. M.; Russo C.; Schmidt W. P.; Stewart J.; Zambrana W.; Zuin V.; Nalá R.; Cumming O.; Brown J. Effects of an Urban Sanitation Intervention on Childhood Enteric Infection and Diarrhea in Maputo, Mozambique: A Controlled before-and-after Trial. eLife 2021, 10, 361 10.7554/eLife.62278. PMC8121544 33835026

[ref17] Capone D.; Berendes D.; Cumming O.; Holcomb D.; Knee J.; Konstantinidis K. T.; Levy K.; Nalá R.; Risk B. B.; Stewart J.; Brown J. Impact of an Urban Sanitation Intervention on Enteric Pathogen Detection in Soils. Environ. Sci. Technol. 2021, 55, 9989–10000. 10.1021/acs.est.1c02168. 34236178 PMC8327413

[ref18] Steinbaum L.; Mboya J.; Mahoney R.; Njenga S. M.; Null C.; Pickering A. J. Effect of a Sanitation Intervention on Soil-Transmitted Helminth Prevalence and Concentration in Household Soil: A Cluster-Randomized Controlled Trial and Risk Factor Analysis. PLoS Neglected Trop. Dis. 2019, 13, e0007180 10.1371/journal.pntd.0007180. PMC6386409 30742614

[ref19] Pickering A. J.; Djebbari H.; Lopez C.; Coulibaly M.; Alzua M. L. Effect of a Community-Led Sanitation Intervention on Child Diarrhoea and Child Growth in Rural Mali: A Cluster-Randomised Controlled Trial. Lancet Glob. Health 2015, 3, e701–e711. 10.1016/S2214-109X(15)00144-8. 26475017

[ref20] Holcomb D. A.; Knee J.; Sumner T.; Adriano Z.; de Bruijn E.; Nalá R.; Cumming O.; Brown J.; Stewart J. R. Human Fecal Contamination of Water, Soil, and Surfaces in Households Sharing Poor-Quality Sanitation Facilities in Maputo, Mozambique. Int. J. Hyg. Environ. Health 2020, 226, 113496 10.1016/j.ijheh.2020.113496. 32135507 PMC7174141

[ref21] Ercumen A.; Pickering A. J.; Kwong L. H.; Mertens A.; Arnold B. F.; Benjamin-Chung J.; Hubbard A. E.; Alam M.; Sen D.; Islam S.; Rahman M. Z.; Kullmann C.; Chase C.; Ahmed R.; Parvez S. M.; Unicomb L.; Rahman M.; Ram P. K.; Clasen T.; Luby S. P.; Colford J. M. Do Sanitation Improvements Reduce Fecal Contamination of Water, Hands, Food, Soil, and Flies? Evidence from a Cluster-Randomized Controlled Trial in Rural Bangladesh. Environ. Sci. Technol. 2018, 52, 12089–12097. 10.1021/acs.est.8b02988. 30256095 PMC6222553

[ref22] Pickering A. J.; Swarthout J.; Mureithi M.; Mboya J.; Arnold B. F.; Wolfe M.; Dentz H. N.; Lin A.; Arnold C. D.; Rao G.; Stewart C. P.; Ram P. K.; Clasen T.; Colford J. M.; Null C. Can Individual and Integrated Water, Sanitation, and Handwashing Interventions Reduce Fecal Contamination in the Household Environment? Evidence from the WASH Benefits Cluster-Randomized Trial in Rural Kenya. bioRxiv 2019, 731992 10.1101/731992.

[ref23] Fuhrmeister E. R.; Ercumen A.; Pickering A. J.; Jeanis K. M.; Crider Y.; Ahmed M.; Brown S.; Alam M.; Sen D.; Islam S.; Kabir M. H.; Islam M.; Rahman M.; Kwong L. H.; Arnold B. F.; Luby S. P.; Colford J. M.; Nelson K. L. Effect of Sanitation Improvements on Pathogens and Microbial Source Tracking Markers in the Rural Bangladeshi Household Environment. Environ. Sci. Technol. 2020, 54, 4316–4326. 10.1021/acs.est.9b04835. 32167305 PMC7144219

[ref24] Haas C. N. Microbial Dose Response Modeling: Past, Present, and Future. Environ. Sci. Technol. 2015, 49, 1245–1259. 10.1021/es504422q. 25545032

[ref25] Emerson P. M.; Lindsay S. W.; Walraven G.; el Faal H.; Bøgh C.; Lowe K.; Bailey R. L. Effect of Fly Control on Trachoma and Diarrhoea. Lancet 1999, 353, 1401–1403. 10.1016/S0140-6736(98)09158-2. 10227221

[ref26] Chavasse D.; Shier R.; Murphy O.; Huttly S.; Cousens S.; Akhtar T. Impact of Fly Control on Childhood Diarrhoea in Pakistan: Community-Randomised Trial. Lancet 1999, 353, 22–25. 10.1016/S0140-6736(98)03366-2. 10023946

[ref27] Cohen D.; Green M.; Block C.; Slepon R.; Ambar R.; Wasserman S. S.; Levine M. M. Reduction of Transmission of Shigellosis by Control of Houseflies (*Musca domestica*). Lancet 1991, 337, 993–997. 10.1016/0140-6736(91)92657-N. 1673210

[ref28] Water and Sanitation for the Urban Poor. An Integrated Approach to Peri-Urban Sanitation and Hygiene in Maputo: Working with City Authorities to Improve Services and Practices; Water and Sanitation for the Urban Poor: Maputo, 2018.

[ref29] Bell M.; Irish S.; Schmidt W. P.; Nayak S.; Clasen T.; Cameron M. Comparing Trap Designs and Methods for Assessing Density of Synanthropic Flies in Odisha, India. Parasites Vectors 2019, 12, 75 10.1186/s13071-019-3324-z. 30732628 PMC6367737

[ref30] Capone D.; Adriano Z.; Berendes D.; Cumming O.; Dreibelbis R.; Holcomb D. A.; Knee J.; Ross I.; Brown J. A Localized Sanitation Status Index as a Proxy for Fecal Contamination in Urban Maputo, Mozambique. PLoS One 2019, 14, e0224333 10.1371/journal.pone.0224333. 31652287 PMC6814227

[ref31] Borchardt M. A.; Boehm A. B.; Salit M.; Spencer S. K.; Wigginton K. R.; Noble R. T. The Environmental Microbiology Minimum Information (EMMI) Guidelines: QPCR and DPCR Quality and Reporting for Environmental Microbiology. Environ. Sci. Technol. 2021, 55, 10210–10223. 10.1021/acs.est.1c01767. 34286966

[ref32] Liu J.; Gratz J.; Amour C.; Kibiki G.; Becker S.; Janaki L.; Verweij J. J.; Taniuchi M.; Sobuz S. U.; Haque R.; Haverstick D. M.; Houpt E. R. A Laboratory-Developed Taqman Array Card for Simultaneous Detection of 19 Enteropathogens. J. Clin. Microbiol. 2013, 51, 472–480. 10.1128/JCM.02658-12. 23175269 PMC3553916

[ref33] Liu J.; Platts-Mills J. A.; Juma J.; Kabir F.; Nkeze J.; Okoi C.; Operario D. J.; Uddin J.; Ahmed S.; Alonso P. L.; Antonio M.; Becker S. M.; Blackwelder W. C.; Breiman R. F.; Faruque A. S. G.; Fields B.; Gratz J.; Haque R.; Hossain A.; Hossain M. J.; Jarju S.; Qamar F.; Iqbal N. T.; Kwambana B.; Mandomando I.; McMurry T. L.; Ochieng C.; Ochieng J. B.; Ochieng M.; Onyango C.; Panchalingam S.; Kalam A.; Aziz F.; Qureshi S.; Ramamurthy T.; Roberts J. H.; Saha D.; Sow S. O.; Stroup S. E.; Sur D.; Tamboura B.; Taniuchi M.; Tennant S. M.; Toema D.; Wu Y.; Zaidi A.; Nataro J. P.; Kotloff K. L.; Levine M. M.; Houpt E. R. Use of Quantitative Molecular Diagnostic Methods to Identify Causes of Diarrhoea in Children: A Reanalysis of the GEMS Case-Control Study. Lancet 2016, 388, 1291–1301. 10.1016/S0140-6736(16)31529-X. 27673470 PMC5471845

[ref34] Baker K. K.; Senesac R.; Sewell D.; Sen Gupta A.; Cumming O.; Mumma J. Fecal Fingerprints of Enteric Pathogen Contamination in Public Environments of Kisumu, Kenya Associated with Human Sanitation Conditions and Domestic Animals. Environ. Sci. Technol. 2018, 52, 10263 10.1021/acs.est.8b01528. 30106283 PMC6557411

[ref35] Capone D.; Berendes D.; Cumming O.; Holcomb D.; Knee J.; Konstantinidis K. T.; Levy K.; Nalá R.; Risk B. B.; Stewart J.; Brown J. Impact of an Urban Sanitation Intervention on Enteric Pathogen Detection in Soils. Environ. Sci. Technol. 2021, 55, 9989 10.1021/acs.est.1c02168. 34236178 PMC8327413

[ref36] Capone D.; Chigwechokha P.; de los Reyes F. L.; Holm R. H.; Risk B. B.; Tilley E.; Brown J. Impact of Sampling Depth on Pathogen Detection in Pit Latrines. PLoS Neglected Trop. Dis. 2021, 15, e0009176 10.1371/journal.pntd.0009176. PMC7954291 33651818

[ref37] Kodani M.; Winchell J. M. Engineered Combined-Positive-Control Template for Real-Time Reverse Transcription-PCR in Multiple-Pathogen-Detection Assays. J. Clin. Microbiol. 2012, 50, 1057–1060. 10.1128/JCM.05987-11. 22170926 PMC3295119

[ref38] Stokdyk J. P.; Firnstahl A. D.; Spencer S. K.; Burch T. R.; Borchardt M. A. Determining the 95% Limit of Detection for Waterborne Pathogen Analyses from Primary Concentration to QPCR. Water Res. 2016, 96, 105–113. 10.1016/j.watres.2016.03.026. 27023926

[ref39] Walker D. I.; McQuillan J.; Taiwo M.; Parks R.; Stenton C. A.; Morgan H.; Mowlem M. C.; Lees D. N. A Highly Specific *Escherichia coli* QPCR and Its Comparison with Existing Methods for Environmental Waters. Water Res. 2017, 126, 101–110. 10.1016/j.watres.2017.08.032. 28930669

[ref40] Capone D.; Bivins A.; Brown J. Producing Ratio Measures of Effect with Quantitative Microbial Risk Assessment. Risk Analysis 2022, 13972 10.1111/risa.13972. PMC9734285 35689350

[ref41] Lindeberg Y. L.; Egedal K.; Hossain Z. Z.; Phelps M.; Tulsiani S.; Farhana I.; Begum A.; Jensen P. K. M. Can *Escherichia coli* Fly? The Role of Flies as Transmitters of E. Coli to Food in an Urban Slum in Bangladesh. Trop. Med. Int. Health 2018, 23, 2–9. 10.1111/tmi.13003. 29121443

[ref42] Delignette-Muller M. L.; Dutang C. Fitdistrplus: An R Package for Fitting Distributions. J. Stat. Softw. 2015, 64, 1–34. 10.18637/jss.v064.i04.

[ref43] Steinbaum L.; Kwong L. H.; Ercumen A.; Negash M. S.; Lovely A. J.; Njenga S. M.; Boehm A. B.; Pickering A. J.; Nelson K. L. Detecting and Enumerating Soil-Transmitted Helminth Eggs in Soil: New Method Development and Results from Field Testing in Kenya and Bangladesh. PLoS Neglected Trop. Dis. 2017, 11, e0005522 10.1371/journal.pntd.0005522. PMC5393894 28379956

[ref44] Capone D.; Bivins A.; Knee J.; Cumming O.; Nalá R.; Brown J. Quantitative Microbial Risk Assessment of Pediatric Infections Attributable to Ingestion of Fecally Contaminated Domestic Soils in Low-Income Urban Maputo, Mozambique. Environ. Sci. Technol. 2021, 55, 1941–1952. 10.1021/acs.est.0c06972. 33472364 PMC7860170

[ref45] Sato M. I. Z.; Galvani A. T.; Padula J. A.; Nardocci A. C.; Lauretto M.; de S.; Razzolini M. T. P.; Hachich E. M. Assessing the Infection Risk of Giardia and Cryptosporidium in Public Drinking Water Delivered by Surface Water Systems in Sao Paulo State, Brazil. Sci. Total Environ. 2013, 442, 389–396. 10.1016/j.scitotenv.2012.09.077. 23178841

[ref46] Wing C.; Simon K.; Bello-Gomez R. A. Designing Difference in Difference Studies: Best Practices for Public Health Policy Research. Annu. Rev. Public Health 2018, 39, 453–469. 10.1146/annurev-publhealth-040617-013507. 29328877

[ref47] Halekoh U.; Højsgaard S.; Yan J. The R Package Geepack for Generalized Estimating Equations. J. Stat. Softw. 2006, 15, 1–11. 10.18637/jss.v015.i02.

[ref48] Schreiner M. Simple Poverty Scorecard Poverty-Assessment Tool Mozambique; Swiss Development Corporation, 2013.

[ref49] Zhang Z.; Mai Y.; Yang M.; Zhang M. Z. Package ‘WebPower’, Basic and Advanced Statistical Power Analysis Version, 2018.

[ref50] Graham-Smith G. S. Observations on the Ways in Which Artificially Infected Flies (Musca domestica) Carry and Distribute Pathogenic and Other Bacteria, 1st ed.; Darling and Son, Ltd.: London, 1910; Vol. 40.

[ref51] Wenyon C. M.; O’connor F. W. An Inquiry Into Some Problems Affecting the Spread and Incidence of Intestinal Protozoal Infections of British Troops and Natives in Egypt, with Special Reference to the Carrier Question, Diagnosis, and Treatment of Amoebic Dysentery, and an Account of Th. J. R. Army Med. Corps 1917, 28, 1 10.1136/jramc-28-02-01.

[ref52] Rusin P.; Maxwell S.; Gerba C. Comparative Surface-to-Hand and Fingertip-to-Mouth Transfer Efficiency of Gram-Positive Bacteria, Gram-Negative Bacteria, and Phage. J. Appl. Microbiol. 2002, 93, 585–592. 10.1046/j.1365-2672.2002.01734.x. 12234341

[ref53] Fongaro G.; Nascimento M. A.; do Rigotto C.; Ritterbusch G.; da Silva A. D.; Esteves P. A.; Barardi C. R. M. Evaluation and Molecular Characterization of Human Adenovirus in Drinking Water Supplies: Viral Integrity and Viability Assays. Virol. J. 2013, 10, 166 10.1186/1743-422X-10-166. 23714224 PMC3686584

[ref54] Olson M. E.; Goh J.; Phillips M.; Guselle N.; McAllister T. A. Giardia Cyst and Cryptosporidium Oocyst Survival in Water, Soil, and Cattle Feces. J. Environ. Qual. 1999, 28, 1991 10.2134/jeq.1999.00472425002800060040x.

[ref55] Amrhein V.; Greenland S.; McShane B. Scientists Rise up against Statistical Significance. Nature 2019, 567, 305–307. 10.1038/d41586-019-00857-9. 30894741

[ref56] Grassi G. B. I Chetognati; Engelmann, 1883.

[ref57] Ercumen A.; Pickering A. J.; Kwong L. H.; Arnold B. F.; Parvez S. M.; Alam M.; Sen D.; Islam S.; Kullmann C.; Chase C.; Ahmed R.; Unicomb L.; Luby S. P.; Colford J. M. Animal Feces Contribute to Domestic Fecal Contamination: Evidence from E. Coli Measured in Water, Hands, Food, Flies, and Soil in Bangladesh. Environ. Sci. Technol. 2017, 51, 8725–8734. 10.1021/acs.est.7b01710. 28686435 PMC5541329

[ref58] De Jesús A. J.; Olsen A. R.; Bryce J. R.; Whiting R. C. Quantitative Contamination and Transfer of *Escherichia coli* from Foods by Houseflies, *Musca domestica* L. (Diptera: Muscidae). Int. J. Food Microbiol. 2004, 93, 259–262. 10.1016/j.ijfoodmicro.2003.12.003. 15135963

[ref59] Collinet-Adler S.; Babji S.; Francis M.; Kattula D.; Premkumar P. S.; Sarkar R.; Mohan V. R.; Ward H.; Kang G.; Balraj V.; Naumova E. N. Environmental Factors Associated with High Fly Densities and Diarrhea in Vellore, India. Appl. Environ. Microbiol. 2015, 81, 6053–6058. 10.1128/AEM.01236-15. 26116684 PMC4551260

[ref60] Songe M.; Hang’ombe B.; Knight-Jones T.; Grace D. Antimicrobial Resistant Enteropathogenic *Escherichia coli* and Salmonella Spp. in Houseflies Infesting Fish in Food Markets in Zambia. Int. J. Environ. Res. Public Health 2017, 14, 21 10.3390/ijerph14010021. PMC5295272 28036049

[ref61] Akter S.; Sabuj A. A. M.; Haque Z. F.; Rahman M. T.; Kafi M. A.; Saha S. Detection of Antibiotic-Resistant Bacteria and Their Resistance Genes from Houseflies. Vet. World 2020, 13, 266–274. 10.14202/vetworld.2020.266-274. 32255968 PMC7096309

[ref62] Khamesipour F.; Lankarani K. B.; Honarvar B.; Kwenti T. E. A Systematic Review of Human Pathogens Carried by the Housefly (*Musca domestica* L.). BMC Public Health 2018, 18, 1049 10.1186/s12889-018-5934-3. 30134910 PMC6104014

[ref63] Holcomb D. A.; Knee J.; Capone D.; Sumner T.; Adriano Z.; Nalá R.; Cumming O.; Brown J.; Stewart J. R. Impacts of an Urban Sanitation Intervention on Fecal Indicators and the Prevalence of Human Fecal Contamination in Mozambique. Environ. Sci. Technol. 2021, 55, 11667–11679. 10.1021/acs.est.1c01538. 34382777 PMC8429117

[ref64] Yoon K. S.; Kwon D. H.; Strycharz J. P.; Hollingsworth C. S.; Lee S. H.; Clark J. M. Biochemical and Molecular Analysis of Deltamethrin Resistance in the Common Bed Bug (Hemiptera: Cimicidae). J. Med. Entomol. 2008, 45, 1092–1101. 10.1603/0022-2585(2008)45[1092:bamaod]2.0.co;2. 19058634

[ref65] Acevedo G. R.; Zapater M.; Toloza A. C. Insecticide Resistance of House Fly, *Musca domestica* (L.) from Argentina. Parasitol. Res. 2009, 105, 489–493. 10.1007/s00436-009-1425-x. 19340457

[ref66] Berendes D. M.; Yang P. J.; Lai A.; Hu D.; Brown J. Estimation of Global Recoverable Human and Animal Faecal Biomass. Nat. Sustain. 2018, 1, 679–685. 10.1038/s41893-018-0167-0. PMC10922008 38464867

[ref67] Hargreaves E. Entomological Notes from Taranto (Italy) with References to Faenza, during 1917 and 1918. Bull. Entomol. Res. 1923, 14, 213–219. 10.1017/S0007485300056261.

[ref68] Hamer D. H.; Ram P. K.; Lawrence J. J.; Osbert N.; Yeboah-Antwi K.; Sabin L. L.; Biemba G. Beliefs, Behaviors, and Perceptions of Community-Led Total Sanitation and Their Relation to Improved Sanitation in Rural Zambia. Am. J. Trop. Med. Hyg. 2016, 94, 553–562. 10.4269/ajtmh.15-0335. 26787149 PMC4775890

[ref69] Sieyro L. Die Hausfliege (*Musca domestica*) Als Überträger von *Entamoeba histolytica* Und Anderen Darmprotozoen. Dtsch. Tropenmed. Ztschr. 1942, 46, 361–372.

[ref70] Capone D.; Berendes D.; Cumming O.; Knee J.; Nalá R.; Risk B. B.; Stauber C.; Zhu K.; Brown J. Analysis of Fecal Sludges Reveals Common Enteric Pathogens in Urban Maputo, Mozambique. Environ. Sci. Technol. Lett. 2020, 7, 889–895. 10.1021/acs.estlett.0c00610. PMC11177333 38881628

[ref71] Bartelt L. A.; Platts-Mills J. A. Giardia: A Pathogen or Commensal for Children in High-Prevalence Settings?. In Current Opinion in Infectious Diseases; Lippincott Williams and Wilkins, 2016; pp 502–507. 10.1097/QCO.0000000000000293. PMC5123436 27479025

[ref72] Lappan R.; Henry R.; Chown S. L.; Luby S. P.; Higginson E. E.; Bata L.; Jirapanjawat T.; Schang C.; Openshaw J. J.; O’Toole J.; Lin A.; Tela A.; Turagabeci A.; Wong T. H. F.; French M. A.; Brown R. R.; Leder K.; Greening C.; McCarthy D. Monitoring of Diverse Enteric Pathogens across Environmental and Host Reservoirs with TaqMan Array Cards and Standard QPCR: A Methodological Comparison Study. Lancet Planet Health 2021, 5, e297–e308. 10.1016/S2542-5196(21)00051-6. 33964239 PMC8116308

[ref73] Haas C. N. Quantitative Microbial Risk Assessment (QMRA) and Molecular Biology-Paths to Integration. Environ. Sci. Technol. 2020, 54, 8539 10.1021/acs.est.0c00664. 32539352

[ref74] Aw T. Environmental Aspects and Features of Critical Pathogen Groups. In Global Water Pathogen Project; Michigan State University, 2019. 10.14321/waterpathogens.2.

[ref75] Rose J.; Jiménez-Cisneros B.; Murphy H. Persistence of Pathogens in Sewage and Other Water Types. In Global Water Pathogen Project; Michigan State University, 2019. 10.14321/waterpathogens.51.

[ref76] Husseini M.; Darboe M. K.; Moore S. E.; Nabwera H. M.; Prentice A. M. Thresholds of Socio-Economic and Environmental Conditions Necessary to Escape from Childhood Malnutrition: A Natural Experiment in Rural Gambia. BMC Med. 2018, 16, 199 10.1186/s12916-018-1179-3. 30382849 PMC6211595

