# Supplementary material for: Urban Onsite Sanitation Upgrades and Synanthropic Flies in Maputo, Mozambique: Effects on Enteric Pathogen Infection Risks
Source: Environ Sci Technol. 2022 Dec 14;57(1):549–60. doi: 10.1021/acs.est.2c06864 (PMC9835884; doi:10.1021/acs.est.2c06864)
Supplement: Supplementary file 1 — es2c06864_si_001.pdf [file es2c06864_si_001.pdf]

Supporting Information

Urban Onsite Sanitation Upgrades and Synanthropic Flies in Maputo, Mozambique: Effects on Enteric Pathogen Infection Risks

Authors: Drew Capone[1,*], Zaida Adriano[2], Oliver Cumming[3], Seth R. Irish[4], Jackie Knee[3], Rassul Nala[5], Joe Brown[6]

1. Department of Environmental and Occupational Health, School of Public Health, Indiana University, Bloomington, Indiana, 47401
2. WE Consult ltd, 177 Rua Tomas Ribeiro, Maputo 1102, Mozambique
3. Department of Disease Control, London School of Hygiene and Tropical Medicine, London, United Kingdom
4. Epidemiology and Public Health Department, Swiss Tropical and Public Health Institute, Kreuzstrasse 2, Allschwil 4123, Switzerland
5. Ministério da Saúde, Instituto Nacional de Saúde Maputo, Maputo, Mozambique
6. Department of Environmental Sciences and Engineering, Gillings School of Public Health, University of North Carolina at Chapel Hill, Chapel Hill, North Carolina 27599

*Corresponding Author: Drew Capone, Email: dscapone@iu.edu, Address: 2719 E 10th St, Bloomington, IN 47408, USA

Supplemental Information: 25 pages, 2 Texts, 2 Figures, and 8 Tables

1. Text S1. Fly Trap Piloting
2. Figure S1. Plots of *E. coli* concentrations
3. Text S2. Fly selection process
4. Table S1. TAC Performance
5. Figure S2. RT-qPCR plots
6. Table S2. MIQE Checklist
7. Table S3. QMRA Parameters
8. Table S4. Post hoc power
9. Table S5. Sensitivity Analysis Parameters

10. Table S6. Fly Counts and Prevalence
11. Table S7. Sensitivity Analysis Results
12. Table S8. Pathogen prevalence comparison

Text S1. Fly trap piloting

We piloted three methods for fly enumeration and capture: baited traps, sticky traps, and a scudder grill[1]. Baited traps were unusable because household animals (mostly cats) would steal the bait. The scudder grill performed well for fly enumeration, but proved logistically challenging for the field workers to carry each day in addition to the other materials needed for anthropometry, stool collection, and environmental sampling. As field workers were required to return to the compound the following day to collect the child's stool sample, the need to pick up sticky fly traps the following day was not a logistical challenge. We choose to use sticky fly traps for fly enumeration and collection based off these piloting experiences.

Figure S1. Plots of *E. coli* concentrations

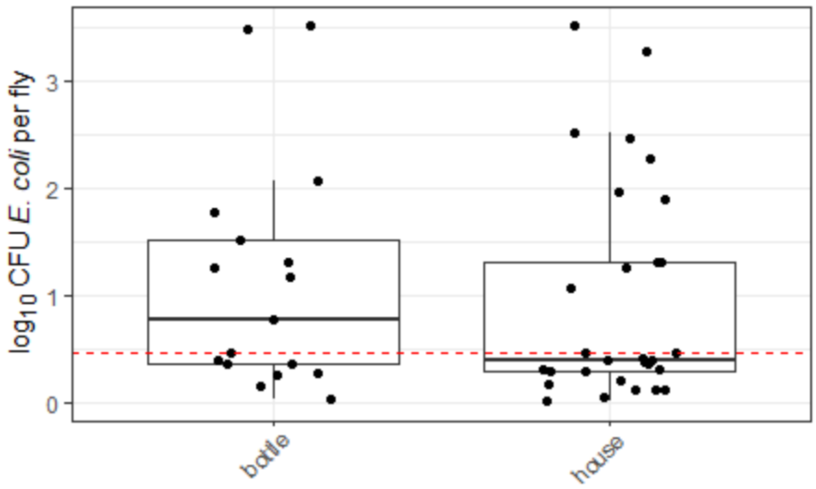

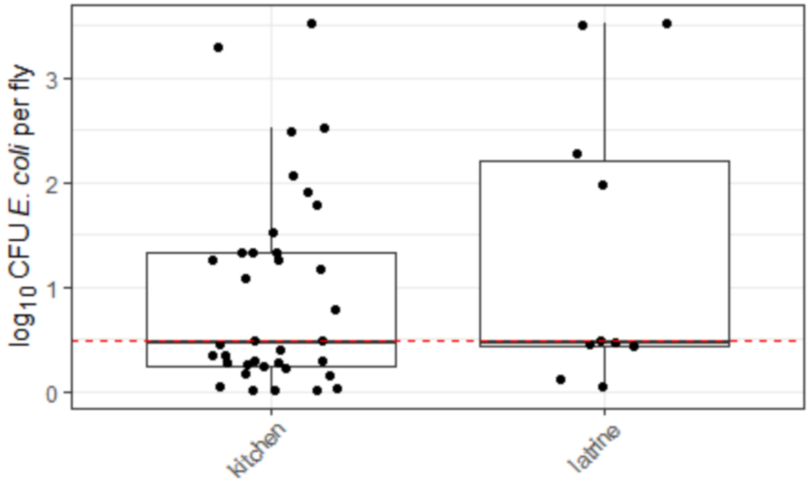

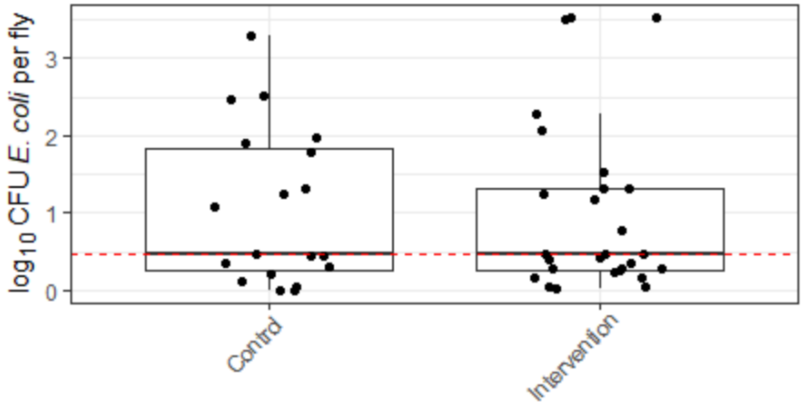

Text S2. Fly selection process

We randomly selected flies from 46 compounds collected at baseline for culture analysis. As the capture of flies were heterogenous, we followed a procedure to select flies. We selected houseflies over bottleflies and flies from the food preparation area over the latrine entrance. Some examples of this procedure are:

1. Compound X
    a. Food preparation area: 1 housefly
    b. Latrine entrance: 0 flies
    c. Result: We selected the housefly from the food preparation area because it was the only fly available
2. Compound Y
    a. Food preparation area: 2 houseflies, 2 bottle flies
    b. Latrine entrance: 0 flies
    c. Result: We randomly selected a housefly from the food preparation area because we selected houseflies over bottleflies
3. Compound Z
    a. Food preparation area: 0 houseflies, 2 bottle flies
    b. Latrine entrance: 2 houseflies, 2 bottle flies
    c. Result: We randomly selected a bottlefly from the food preparation area because we selected flies from the food preparation area over the latrine entrance, and there were no houseflies available from the food preparation area

Figure S2. RT-qPCR plots

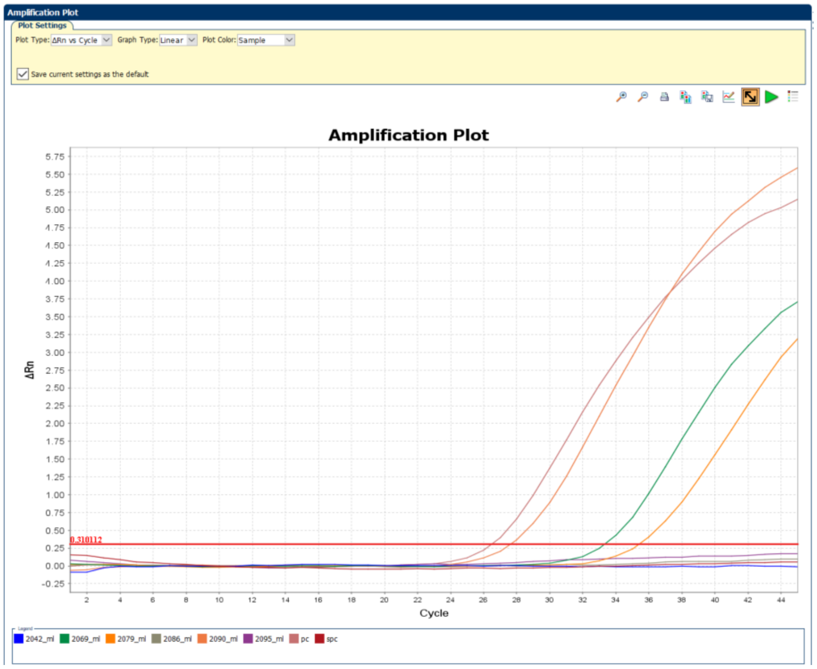

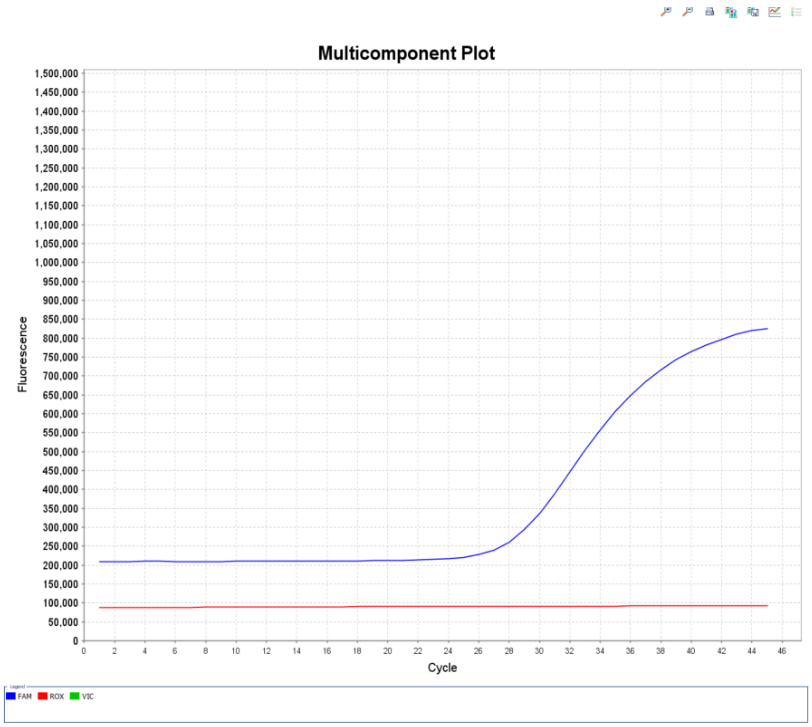

Table S1. TAC Performance and Information

Name	Gene	R^2	efficiency	y-intercept	95% LOD[9] (gene copies / fly)*	Reference	Length	Accession #
adenovirus	Hexon b	0.994	98%	38.0	833	[10]	131	KX868289.2
Ancylostoma duodenale	ITS2	0.994	93%	39.9	1833	[11]	72	MK271367.1
Ascaris lumbricoides	ITS1	0.992	94%	39.3	22334	[11]	88	LK872896.1
astrovirus	Capsid	0.997	99%	36.6	1000	[10]	172	MG921619.1
Campylobacter jejuni & *coli*	cadF	0.999	91%	38.5	6833	[10]	221	CP044164.1
Clostridium difficile	tcdB	1.000	96%	37.9	833	[10]	87	CP028526.1
Cryptosporidium parvum	LIB13	0.999	93%	42.2	167	[11]	166	B78618.1
Cryptosporidium hominis	LIB13	0.999	96%	39.4	2000	[11]	169	AF190627.1
Enteroaggregative *E. coli*	aaiC	0.997	99%	37.6	3167	[10]	215	CP042953.1
Enteroaggregative *E. coli*	aatA	0.987	104%	37.6	8500	[10]	237	MG564313.1
Entamoeba histolytica	18S	0.993	97%	41.0	7833	[10]	174	MK332025.1
enterovirus	5'UTR	0.999	97%	36.5	1000	[10]	148	AF538841.1
Enteropathogenic *E. coli*	bfpA	0.994	98%	38.1	833	[10]	110	CP042948.1
Enteropathogenic *E. coli*	eae	0.995	97%	39.4	833	[10]	102	CP032811.1
Enterotoxigenic *E. coli*	LT	0.988	90%	36.9	22334	[10]	62	CP042619.1
Enterotoxigenic *E. coli*	STh	0.996	96%	38.2	1833	[10]	147	CP025855.1
Enterotoxigenic *E. coli*	STp	0.994	98%	37.9	1833	[10]	136	CP042629.1
Giardia spp.	18S	0.999	96%	39.4	1833	[10]	63	MK993323.1
Shigella spp./Enteroinvasive *E. coli*	ipaH	0.999	100%	38.7	1833	[10]	64	CP044197.1
Necator americanus	ITS2	0.994	96%	40.6	21167	[11]	101	MH665842.1
norovirus GI	ORF1-ORF2	1.000	95%	35.6	1000	[11]	85	MN448477.1
norovirus GII	ORF1-ORF2	0.999	97%	35.3	1000	[11]	98	MN453358.1
rotavirus A	NSP3	0.998	89%	38.1	167	[10]	87	LC260307.1
Salmonella spp.	invA	0.996	95%	40.1	21167	[10]	95	CP055130.1
sapovirus I/II/IV	RdRp	0.998	97%	34.3	167	[10]	104	MK111630.1

sapovirus V	RdRp	0.998	97%	35.8	167	10	107	MK291480.1
shiga toxin producing *E. coli*	stx1	0.998	97%	36.9	7833	10	132	CP032805.1
shiga toxin producing *E. coli*	stx2	0.997	97%	36.0	7833	10	93	CP032811.1
Trichuris trichiura†	18S	0.999	101%	40.5	2000	10	76	AB699092.1
Vibrio cholerae‡	hlyA	0.999	96%	39.5	1833	11	102	CP026532.1

*Note: A 95% LOD of 167 gene copies per fly corresponds to a 95% LOD of 1 gene copy per reaction well.

†May cross-react with *Trichuris vulpis*

‡May cross-react with other *Vibrio* spp. that contain the hlyA gene

Table S2. MIQE Checklist

Group	Item	Response
Experimental Design	Definition of experimental and control groups	The intervention group received the onsite sanitation intervention, and the control group did not.
	Number within each group	Baseline: Control (n=56), Intervention (n=34)<br><br>12-month: Control (n=53), Intervention (n=33)
	Assay carried out by the core or investigator's laboratory?	Assays were carried out by the Brown Lab at UNC-Chapel Hill.
	Acknowledgment of authors' contributions	DC: Conceptualization, Formal analysis, Investigation, Writing - Original Draft<br><br>ZA: Methodology, Supervision, Writing - Review & Editing<br><br>OC: Methodology, Funding acquisition, Writing - Review & Editing<br><br>SI: Methodology, Writing - Review & Editing<br><br>JK: Methodology, Writing - Review & Editing<br><br>RN: Supervision, Resources, Writing - Review & Editing<br><br>JB: Conceptualization, Funding acquisition, Writing - Review & Editing
Sample	Description	Samples were houseflies and green bottle flies caught at latrine entrances and food preparation areas among low-income informal settlements in Maputo, Mozambique.

	Volume/mass of sample processed	Fly Mass Summary (mg):<br>Mean = 9.8<br>Median = 8.90 (IQR= 4.80, 13)<br>Min = 0.40<br>Max = 52.90
	Processing procedure	Methods described in the main text.
	If frozen, how and how quickly?	Samples were frozen at -80C within six hours of collection.
	If fixed, with what and how quickly?	Not fixed.
	Sample storage conditions and duration	Samples remained at -80C approximately five years before analysis.
Nucleic acid extraction	Procedure and/or instrumentation	Methods described in the main text.
	Name of kit and details of any modifications	Methods described in the main text.
	Source of additional reagents used	Methods described in the main text.
	Details of DNase or RNase treatment	Not treated
	Contamination assessment (DNA or RNA)	One extraction negative control was included per day of extractions.
	Nucleic acid quantification	Nucleic acid was not quantified since most of the nucleic acid would have been from the fly itself.
	Instrument and method	Manual extractions were performed in a biological safety cabinet.
	Inhibition testing (Cq dilutions, spike, or other)	Inhibition was monitored using spike in control: RNA bacteriophage MS2 (ATCC, Manassas, VA) and a synthetic DNA sequence (IDT, Coralville, IA)

Reverse transcription	Complete reaction conditions	One-step reverse transcription
	Amount of RNA and reaction volume	Reaction Volume = 1.5 µL
	Reverse transcriptase and concentration	ArrayScript™ Reverse Transcriptase
	Temperature and time	45°C for 20 minutes
	Manufacturer of reagents and catalogue numbers	Applied Biosystems, AgPath-ID™ One-Step RT-PCR Reagents Catalog number: 4387391
qPCR target information	Gene symbol	Provided in Table S1
	Sequence accession number	Provided in Table S1
	Amplicon length	Provided in Table S1
	In silico specificity screen (BLAST, and so on)	We BLASTed all assays to confirm specificity before ordering the custom TAC. See the note below Table S1.
qPCR oligonucleotides	Primer sequences	Primer sequences were taken from Table 1 in Liu *et al*. 2013[10] and Table S2 in Liu *et al*. 2016[11].
	Probe sequences	Primer sequences were taken from Table 1 in Liu *et al*. 2013[10] and Table S2 in Liu *et al*. 2016[11].
	Location and identity of any modifications	No modifications
	Manufacturer of oligonucleotides	ThermoFisher Scientific
qPCR protocol	Complete reaction conditions	45°C for 20 min and 95°C for 10 min, followed by 45 cycles of 95°C for 15 s and 60°C for 1 min
	Reaction volume and amount of cDNA/DNA	100µL reactions were prepared, containing 60uL of mastermix and 40µL of template. This corresponds

		to 0.6µL of template and 0.9µL of mastermix per reaction well.
	Primer, (probe), Mg2+, and dNTP concentrations	All assays contained the same concentrations of primers (900 nanomolar) and probe (250 nanomolar). The Mg2+ and dNTP concentrations are not listed in the in the User Guide.
	Polymerase identity and concentration	AmpliTaq Gold™ polymerase
	Buffer/kit identity and manufacturer	AgPath-ID™ One-Step RT-PCR Reagents
	Additives (SYBR Green I, DMSO, and so forth)	No additives
	Manufacturer of plates/tubes and catalog number	ThermoFisher Scientific
	Complete thermocycling parameters	45°C for 20 min and 95°C for 10 min, followed by 45 cycles of 95°C for 15 s and 60°C for 1 min
	Reaction setup (manual/robotic)	Manual set-up in a disinfected dead air box (10% bleach with fifteen minutes of contact time, UV for fifteen minutes, and a final cleaning step with 70% ethanol)
	Manufacturer of qPCR instrument	ThermoFisher Scientfic
qPCR validation	Evidence of optimization (from gradients)	See Liu *et al*. 2013[10] and Liu *et al*. 2016[11]
	Specificity (gel, sequence, melt, or digest)	See Liu *et al*. 2013[10] and Liu *et al*. 2016[11].
	Calibration curves with slope and y intercept	See Table S1
	PCR efficiency calculated from slope	See Table S1

	r2 of calibration curve	See Table S1
	Evidence for LOD	See Table S1
Data analysis	qPCR analysis program (source, version)	QuantStudio Real-Time PCR Software V1.2 CDC
	Method of Cq determination	Manual thresholding
	Results for NTCs	We observed no amplification before at Ct of 40 in our two PCR negative controls.<br><br>Among the 12 negative extraction controls, we observed no amplification before a Ct of 40.
	Description of normalization method	Normalized per individual fly
	Power analysis	See Power Analysis section in the methods.
	Statistical methods for results significance	Methods described in the main text
	Software (source, version)	R Studio V2.2.2

78 Table S3. QMRA Parameters

Model variable	Parameter/method used	Reference
**Hazard Identification**		
Selected pathogens	qPCR	Liu et al. 2016[11]
**Exposure Assessment**		
Non-detect values (gene copies / fly)	U(0, 95% LOD)	Canales *et al*. 2018[2]
Fly mass (mg)	LN(1.9, 0.91)<br>*truncated at 50mg	MLE, this study
Pathogen transfer efficiency from fly to food	LN(-4.2, 0.89)	De Jesús et al. 2004[12]; MLE, this study
Fly landings on food per year	52	Assumption
Contaminated food consumed by individual	100%	Assumption
Pathogen die-off after deposition but before consumption	0%	Assumption
**Infectious unit**		
Culturable *E. coli* in flies (log_{10} CFU / fly)	Median: 0.45<br>Mean: 1.0<br>SD: 1.1	This study
ybbW in flies (log_{10} gene copies / fly)	Median: 3.0<br>Mean: 3.6<br>SD: 1.6	This study
Log_{10} ratio of infectious cysts/CFUs/viruses to gene copies of target pathogen	N(-2.53, 1.34)<br><br>Distribution from ratios of CFUs *E. coli* to *ybbW* (truncated at 1)	MLE, this study
Probability of *Trichuris* ova viability	Binom(0.75)	Steinbaum *et al*. 2017 and 2019[13,14]
**Dose Harmonization**		
beta-giardin gene copies per *Giardia* spp. cyst	16	Bernander *et al*. 2001[15]
ST/LT gene copies per ETEC CFU	U(1,16)	Lothigius et al. 2008[16]; Youmans et al. 2014[17]
eae/bfpA gene copies per EPEC CFU	5.6	Corsi et al. 2015
aaiC/aatA gene copies per EAEC CFU	U(1,16)	Lothigius et al. 2008[16]; Youmans et al. 2014[17]
Hexon gene copies per adenovirus	700	Kundu et al. 2013[18]

18S gene copies per *Trichuris trichiura* ova	U(42, 25000)<br>(This data does not exist for *Trichuris*, so we used the reported range for *Ascaris*.)	Pecson et al. 2006[19]
ybbW gene copies per *E. coli* genome	1	Walker *et al*. 2017[20]
**Dose-Response**		
Giardia duodenalis dose-response parameter, k, exponential model	LN (0.0198, 0.0066)	Rose *et al*. 1991[21]
ETEC dose-response parameters, alpha, N50, approximate beta-Poisson model	log α<br>N(-1.123, 0.1653)<br>log N_{50}<br>N(6.230, 0.643)	CAMRA 2022[22]
EPEC dose-response dose response parameters, alpha, beta, exact beta-Poisson model	α = 0.0373<br>β = 39.71	Teunis *et al*. 2008[23]
EAEC dose-response dose response parameter, k, exponential	α = 0.0373<br>β = 39.71<br>(This data does not exist for EAEC, so we used the same dose response as EPEC.)	Teunis *et al*. 2008[23]
Adenovirus dose-response dose response parameters, alpha, beta, exact beta-Poisson model	log α<br>N(0.708, 0.454)<br>log β<br>N(0.447, 0.358)	Teunis et al. 2016[24]
Trichuris trichiura, alpha, N50, approximate beta-Poisson model<br>Gene copies	α = 0.104<br>N_{50} = 859<br>(This data does not exist for *Trichuris*, so we used the reported dose response for *Ascaris*.)	Navarro et al. 2009[25]

Note: LN = lognormal (mean, sd); N = normal (mean, sd); U = Uniform (min, max); Binom = Binomial(probability). Values correspond to the inputs used for the *rlnorm*, *rnorm*, and *rbinom* functions in R

Table S4. Post hoc power

Pathogen	Observed Power
Giardia	52%
Enteropathogenic *E. coli*	<1%
Enterotoxigenic *E. coli*	9.0%
Enteroaggregative *E. coli*	36%
adenovirus	49%
Trichuris	16%

Note: Post hoc power calculation performed using the *WebPower* package in R

Table S5. Sensitivity analysis parameters

Parameter	Modeled value	Sensitivity analysis value	Reference
Limit of detection	Imputed from 0 to the 95% LOD	Theoretical limit of detection (i.e., one gene copy per PCR reaction)	Canales *et al*. 2018[2]
Viability	Stochastic ratio of culturable *E. coli* to gene copies of *E. coli*	*Giardia*: Uniform distribution from 0% to 10%. adenovirus: 0.1%	*Giardia*: Olson *et al.* 1999[3] adenovirus: Fongaro *et al*. 2013[4]
Transfer efficiency	Stochastic ratio of the mass of a fly speck (0.1 mg) to the distribution of fly mass observed	3.2%*	Fly Regurgitate/Feces: Graham-Smith 1910[5] and Wenyon and O'Connor 1917[6] Fly Body to Food: Rusin et al. 2002[7]
Fly landings on food immediately before consumption	Once per week	Twice per week	Lindeberg *et al*. 2017[8]

*We estimated a transfer efficiency of 3.2% per fly landing using the following parameters. First, Pava-Ripoll et al. 2015 estimated that 80% of pathogens are present in the fly alimentary canal and the remaining 20% are present on the fly body. We used Graham-Smith 1910[5] and Wenyon and O'Connor 1917[6]to estimate that one fly speck (regurgitate or feces) represents approximately 1/25th of the contents of the fly alimentary canal. Then we used Rusin *et al*. 2002 to estimate the transfer of pathogens from the fly body to food (i.e., 0.1%). We then solved the following equation, $\left(0.80 * \frac{1}{25}\right) + (0.20 * 0.001)$, to estimate a transfer efficiency of 3.2%.

Table S6. Fly Counts and Prevalence

Phase	Arm	Location	Mean Number of Flies Caught
Baseline	Control	Latrine	3.7
	Intervention	Latrine	5.8
	Control	Food Preparation	9.7
	Intervention	Food Preparation	13
12-month follow-up	Control	Latrine	2.5
	Intervention	Latrine	0.8
	Control	Food Preparation	2.1
	Intervention	Food Preparation	2.4
			Prevalence of ≥1 fly caught
Baseline	Control	Latrine	46%
	Intervention	Latrine	54%
	Control	Food Preparation	76%
	Intervention	Food Preparation	85%
12-month follow-up	Control	Latrine	49%
	Intervention	Latrine	20%
	Control	Food Preparation	31%
	Intervention	Food Preparation	44%

Table S7. Sensitivity Analysis Results

<u>Non detects set to theoretical LOD</u>

		Pathogen Prevalence in Flies		Mean Annual Risk of Infection (Minimum, Maximum)		RR (95% CI)*	aRR (95% CI)
Pathogen	Trial Arm	Baseline	12-month	Baseline	12-month		
Pooled infection risk (hypothetical pathogen)	Control	NA†		NA†		0.76 (0.42, 1.4)	0.75 (0.42, 1.4)
	Intervention						
enteropathogenic *E. coli*	Control	25% (14/56)	15% (8/53)	17% (1.6%, >99%)	12% (1.6%, >99%)	1.2 (0.41, 3.7)	1.5 (0.51, 4.7)
	Intervention	24% (8/34)	21% (7/33)	19% (1.6%, 95%)	16% (1.6%, >99%)		
adenovirus	Control	21% (12/56)	7.6% (4/53)	15% (4.2%, 98%)	10% (4.1%, >99%)	**0.38 (0.15, 0.96)**	**0.32 (0.14, 0.97)**
	Intervention	27% (9/34)	0% (0/33)	19% (4.4%, >99%)	5.0% (4.3%, 5.7%)		
enteroaggregative *E. coli*	Control	18% (10/56)	17% (9/53)	12% (1.5%, >99%)	11% (1.4%, >99%)	0.49 (0.14, 1.7)	0.43 (0.13, 1.4)
	Intervention	27% (9/34)	15% (5/33)	21% (1.3%, >99%)	9.5% (1.4%, 81%)		
enterotoxigenic *E. coli*	Control	16% (9/56)	13% (7/53)	18% (6.9%, >99%)	16% (7.9%, 91%)	0.83 (0.49, 1.4)	0.80 (0.50, 1.3)
	Intervention	24% (8/34)	9.1% (3/33)	20% (6.1%, 76%)	11% (8.4%, 55%)		
Trichuris trichiura	Control	13% (7/56)	5.7% (3/53)	4.1% (2.3%, >99%)	1.1% (0.2%, 18%)	0.21 (0.03, 1.5)	**0.12 (0.03, 0.48)**
	Intervention	5.9% (2/34)	0% (0/33)	4.1% (0.2%, >99%)	0.3% (0.2%, 0.3%)		
Giardia	Control	7.1% (4/56)	7.6% (4/53)	7.3% (0.2%, >99%)	6.7% (1.0%, >99%)	**0.13 (0.03, 0.52)**	**0.19 (0.05, 0.80)**
	Intervention	15% (5/34)	0% (0/33)	13% (1.0%, >99%)	1.3% (1.1%, 1.6%)		

<u>Giardia and Adenovirus viability taken from the literature</u>

adenovirus	Control	21% (12/56)	7.6% (4/53)	0.6% (0.1%, 4.8%)	1.8% (0.2%, 49%)	**0.10 (0.03, 0.43)**	**0.08 (0.02, 0.42)**
	Intervention	27% (9/34)	0% (0/33)	0.9% (0.1%, 8.5%)	0.3% (0.25, 0.3%)		
Giardia	Control	7.1% (4/56)	7.6% (4/53)	11% (5.2%, >99%)	11% (5.0%, >99%)	**0.38 (0.15, 0.92)**	0.46 (0.20, 1.1)
	Intervention	15% (5/34)	0% (0/33)	18% (5.4%, >99%)	5.7% (5.2%, 6.2%)		

<u>Transfer Efficiency (3.2%)</u>

		Pathogen Prevalence in Flies		Mean Annual Risk of Infection (Minimum, Maximum)		RR (95% CI)*	aRR (95% CI)
Pathogen	Trial Arm	Baseline	12-month	Baseline	12-month		
Pooled infection risk (hypothetical pathogen)	Control	NA†		NA†		0.85 (0.58, 1.2)	0.84 (0.58, 1.2)
	Intervention						
enteropathogenic *E. coli*	Control	25% (14/56)	15% (8/53)	21% (2.7%, >99%)	15% (2.7%, >99%)	1.3 (0.47, 3.5)	1.6 (0.58, 4.4)
	Intervention	24% (8/34)	21% (7/33)	22% (2.7%, 99%)	19% (2.8%, >99%)		
adenovirus	Control	21% (12/56)	7.6% (4/53)	38% (18%, >99%)	34% (21%, >99%)	0.74 (0.54, 1.0)	0.73 (0.52, 1.0)
	Intervention	27% (9/34)	0% (0/33)	43% (14%, >99%)	28% (22%, 37%)		
enteroaggregative *E. coli*	Control	18% (10/56)	17% (9/53)	14% (2.3%, >99%)	14% (2.9%, >99%)	0.59 (0.20, 1.8)	0.52 (0.18, 1.5)
	Intervention	27% (9/34)	15% (5/33)	23% (2.6%, >99%)	13% (2.3%, 92%)		
enterotoxigenic *E. coli*	Control	16% (9/56)	13% (7/53)	23% (12%, >99%)	20% (12%, 94%)	0.85 (0.55, 1.3)	0.83 (0.55, 1.2)
	Intervention	24% (8/34)	9.1% (3/33)	25% (12%, 82%)	18% (12%, 64%)		
Trichuris trichiura	Control	13% (7/56)	5.7% (3/53)	5.2% (0.4%, >99%)	1.7% (0.4%, 27%)	0.24 (0.04, 1.5)	**0.14 (0.04, 0.53)**
	Intervention	5.9% (2/34)	0% (0/33)	4.6% (0.4%, >99%)	0.4% (0.4%, 0.5%)		
Giardia	Control	7.1% (4/56)	7.6% (4/53)	17% (9.8%, >99%)	16% (9.5%, >99%)	0.53 (0.28, 1.0)	0.61 (0.34, 1.1)
	Intervention	15% (5/34)	0% (0/33)	23% (9.2%, >99%)	11% (8.9%, 13%)		

<u>Flies Land Twice Weekly on Food</u>

		Pathogen Prevalence in Flies		Mean Annual Risk of Infection (Minimum, Maximum)		RR (95% CI)*	aRR (95% CI)
Pathogen	Trial Arm	Baseline	12-month	Baseline	12-month		
Pooled infection risk (hypothetical pathogen)	Control	NA†		NA†		0.85 (0.58, 1.2)	0.84 (0.58, 1.2)
	Intervention						
enteropathogenic *E. coli*	Control	25% (14/56)	15% (8/53)	23% (3.4%, >99%)	16% (3.4%, >99%)	1.3 (0.50, 3.4)	1.6 (0.61, 4.3)
	Intervention	24% (8/34)	21% (7/33)	24% (3.4%, >99%)	21% (3.5%, >99%)		
adenovirus	Control	21% (12/56)	7.6% (4/53)	49% (23% >99%)	45% (32%, >99%)	0.86 (0.68, 1.1)	0.85 (0.67, 1.1)
	Intervention	27% (9/34)	0% (0/33)	54% (23%, >99%)	42% (33%, 59%)		
enteroaggregative *E. coli*	Control	18% (10/56)	17% (9/53)	16% (3.3%, >99%)	15% (3.1%, >99%)	0.64 (0.22, 1.8)	0.57 (0.20, 1.6)
	Intervention	27% (9/34)	15% (5/33)	25% (3.2%, >99%)	15% (3.3%, 97%)		

enterotoxigenic *E. coli*	Control	16% (9/56)	13% (7/53)	32% (21%, >99%)	30% (18%, >99%)	0.88 (0.62, 1.2)	0.87 (0.64, 1.2)
	Intervention	24% (8/34)	9.1% (3/33)	35% (19%, 93%)	28% (21%, 81%)		
Trichuris trichiura	Control	13% (7/56)	5.7% (3/53)	6.1% (0.5%, >99%)	2.2% (0.5%, 34%)	0.27 (0.05, 1.6)	**0.15 (0.04, 0.57)**
	Intervention	5.9% (2/34)	0% (0/33)	5.0% (0.5%, >99%)	0.6% (0.5%, 0.6%)		
Giardia	Control	7.1% (4/56)	7.6% (4/53)	20% (12%, >99%)	19% (11%, >99%)	0.58 (0.33, 1.0)	0.65 (0.38, 1.1)
	Intervention	15% (5/34)	0% (0/33)	26% (12%, >99%)	14% (12%, 16%)		

Table S8. Pathogen prevalence comparison

No.	Pathogen	Stool (n=95)	Sludge (n=95)	Soil (n=179)	Flies (n=176)
**Bacteria**					
**1**	EAEC	67%	82%	46%	19%
**2**	*Shigella*/EIEC	51%	76%	23%	4.0%
**3**	ETEC (*ST*/*LT*)	38%	56%	28%	15%
**4**	EPEC	34%	39%	17%	21%
**5**	STEC (*stx1*/*stx2*)	6.3%	15%	2.8%	1.7%
**6**	*Salmonella*	6.3%	8.4%	5.0%	0.0%
7	*Campylobacter jejuni*/*coli*	5.3%	4.2%	9.5%	1.1%
8	*C. difficile*	3.4%	7.4%	14.5%	0.0%
9	*Vibrio cholerae*	0%	1.1%	0%	2.8%
10	*Yersinia* spp.	0%	2.1%	4%	NA
**Viruses**					
1	Sapovirus I/II/IV/V	12%	47%	5%	0.6%
2	Norovirus GI/GII	11%	58%	4.5%	4.0%
3	Astrovirus	8.4%	63%	26.3%	1.7%
4	Adenovirus 40/41	4.2%	44%	19.6%	14%*
5	Rotavirus A	1.1%	8.4%	7.8%	3.4%
**Protozoa**					
**1**	*Giardia duodenalis*	64%	86%	36%	7.4%
**2**	*Cryptosporidium parvum*	12%	24%	6%	0%
**3**	*Entamoeba histolytica*	1.1%	12%	1.7%	1.1%
**STHs**					
1	*Trichuris trichiuria*	42%	65%	23%	6.8%
2	*Ascaris lumbricoides*	32%	88%	63%	0.6%

*This assay was pan-adenovirus and not specific to 40/41

References

(1) Wolfe, M. K.; Dentz, H. N.; Achando, B.; Mureithi, M.; Wolfe, T.; Nul, C.; Pickering, A. J. Adapting and Evaluating a Rapid, Low-Cost Method to Enumerate Flies in the Household Setting. *American Journal of Tropical Medicine and Hygiene* **2017**, *96* (2), 449–456. https://doi.org/10.4269/ajtmh.16-0162.

(2) Canales, R. A.; Wilson, A. M.; Pearce-Walker, J. I.; Verhougstraete, M. P.; Reynolds, K. A. Methods for Handling Left-Censored Data in Quantitative Microbial Risk Assessment. *Appl Environ Microbiol* **2018**, *84* (20). https://doi.org/10.1128/AEM.01203-18.

(3) Olson, M. E.; Goh, J.; Phillips, M.; Guselle, N.; McAllister, T. A. Giardia Cyst and Cryptosporidium Oocyst Survival in Water, Soil, and Cattle Feces. *Journal of Environment Quality* **1999**, *28* (6), 1991. https://doi.org/10.2134/jeq1999.00472425002800060040x.

(4) Fongaro, G.; Nascimento, M. A. do; Rigotto, C.; Ritterbusch, G.; da Silva, A. D.; Esteves, P. A.; Barardi, C. R. M. Evaluation and Molecular Characterization of Human Adenovirus in Drinking Water Supplies: Viral Integrity and Viability Assays. *Virol J* **2013**, *10* (1), 166. https://doi.org/10.1186/1743-422X-10-166.

(5) Graham-Smith, G. S. *Observations on the Ways in Which Artificially Infected Flies (Musca Domestica) Carry and Distribute Pathogenic and Other Bacteria*, 1st. Edition.; Darling and Son, Ltd.: London, 1910; Vol. 40.

(6) Wenyon, C. M.; O'connor, F. W. An Inquiry Into Some Problems Affecting the Spread and Incidence of Intestinal Protozoal Infections of British Troops and Natives in Egypt, with Special Reference to the Carrier Question, Diagnosis, and Treatment of Amoebic Dysentery, and an Account of Th. *J R Army Med Corps* **1917**, *28* (5).

(7) Rusin, P.; Maxwell, S.; Gerba, C. Comparative Surface-to-Hand and Fingertip-to-Mouth Transfer Efficiency of Gram-Positive Bacteria, Gram-Negative Bacteria, and Phage. *J Appl Microbiol* **2002**, *93* (4), 585–592. https://doi.org/10.1046/j.1365-2672.2002.01734.x.

(8) Lindeberg, Y. L.; Egedal, K.; Hossain, Z. Z.; Phelps, M.; Tulsiani, S.; Farhana, I.; Begum, A.; Jensen, P. K. M. Can Escherichia Coli Fly? The Role of Flies as Transmitters of E. Coli to Food in an Urban Slum in Bangladesh. *Tropical Medicine & International Health* **2018**, *23* (1), 2–9. https://doi.org/10.1111/tmi.13003.

(9) Stokdyk, J. P.; Firnstahl, A. D.; Spencer, S. K.; Burch, T. R.; Borchardt, M. A. Determining the 95% Limit of Detection for Waterborne Pathogen Analyses from Primary Concentration to QPCR. *Water Res* **2016**, *96*, 105–113. https://doi.org/10.1016/j.watres.2016.03.026.

(10) Liu, J.; Gratz, J.; Amour, C.; Kibiki, G.; Becker, S.; Janaki, L.; Verweij, J. J.; Taniuchi, M.; Sobuz, S. U.; Haque, R.; Haverstick, D. M.; Houpt, E. R. A Laboratory-Developed Taqman Array Card for Simultaneous Detection of 19 Enteropathogens. *J Clin Microbiol* **2013**, *51* (2), 472–480. https://doi.org/10.1128/JCM.02658-12.

(11) Liu, J.; Gratz, J.; Amour, C.; Nshama, R.; Walongo, T.; Maro, A.; Mduma, E.; Platts-Mills, J.; Boisen, N.; Nataro, J.; Haverstick, D. M.; Kabir, F.; Lertsethtakarn, P.; Silapong, S.; Jeamwattanalert, P.; Bodhidatta, L.; Mason, C.; Begum, S.; Haque, R.; Praharaj, I.; Kang, G.; Houpt, E. R. Optimization of Quantitative PCR Methods for Enteropathogen Detection. *PLoS One* **2016**, *11* (6), e0158199. https://doi.org/10.1371/journal.pone.0158199.

(12) De Jesús, A. J.; Olsen, A. R.; Bryce, J. R.; Whiting, R. C. Quantitative Contamination and Transfer of Escherichia Coli from Foods by Houseflies, Musca Domestica L. (Diptera: Muscidae). https://doi.org/10.1016/j.ijfoodmicro.2003.12.003.

(13) Steinbaum, L.; Mboya, J.; Mahoney, R.; Njenga, S. M.; Null, C.; Pickering, A. J. Effect of a Sanitation Intervention on Soil-Transmitted Helminth Prevalence and Concentration in Household Soil: A Cluster-Randomized Controlled Trial and Risk Factor Analysis. *PLoS Negl Trop Dis* **2019**, *13* (2), e0007180. https://doi.org/10.1371/journal.pntd.0007180.

(14) Steinbaum, L.; Kwong, L. H.; Ercumen, A.; Negash, M. S.; Lovely, A. J.; Njenga, S. M.; Boehm, A. B.; Pickering, A. J.; Nelson, K. L. Detecting and Enumerating Soil-Transmitted Helminth Eggs in Soil: New Method Development and Results from Field Testing in Kenya and Bangladesh. *PLoS Negl Trop Dis* **2017**, *11* (4), e0005522. https://doi.org/10.1371/journal.pntd.0005522.

(15) Bernander, R.; Palm, J. E. D.; Svärd, S. G. Genome Ploidy in Different Stages of the Giardia Lamblia Life Cycle. *Cell Microbiol* **2001**, *3* (1), 55–62. https://doi.org/10.1046/j.1462-5822.2001.00094.x.

(16) Lothigius, Å.; Janzon, A.; Begum, Y.; Sjöling, Å.; Qadri, F.; Svennerholm, A.-M.; Bölin, I. Enterotoxigenic Escherichia Coli Is Detectable in Water Samples from an Endemic Area by Real-Time PCR. *J Appl Microbiol* **2008**, *104* (4), 1128–1136. https://doi.org/10.1111/j.1365-2672.2007.03628.x.

(17) Youmans, B. P.; Ajami, N. J.; Jiang, Z.-D.; Petrosino, J. F.; DuPont, H. L.; Highlander, S. K. Development and Accuracy of Quantitative Real-Time Polymerase Chain Reaction Assays for Detection and Quantification of Enterotoxigenic Escherichia Coli (ETEC) Heat Labile and Heat Stable Toxin Genes in Travelers' Diarrhea Samples. *Am J Trop Med Hyg* **2014**, *90* (1), 124–132. https://doi.org/10.4269/ajtmh.13-0383.

(18) Kundu, A.; McBride, G.; Wuertz, S. Adenovirus-Associated Health Risks for Recreational Activities in a Multi-Use Coastal Watershed Based on Site-Specific Quantitative Microbial Risk Assessment. *Water Res* **2013**, *47* (16), 6309–6325. https://doi.org/10.1016/j.watres.2013.08.002.

(19) Pecson, B. M.; Barrios, J. A.; Johnson, D. R.; Nelson, K. L. A Real-Time PCR Method for Quantifying Viable Ascaris Eggs Using the First Internally Transcribed Spacer Region of Ribosomal DNA. *Appl Environ Microbiol* **2006**, *72* (12), 7864–7872. https://doi.org/10.1128/AEM.01983-06.

(20) Walker, D. I.; McQuillan, J.; Taiwo, M.; Parks, R.; Stenton, C. A.; Morgan, H.; Mowlem, M. C.; Lees, D. N. A Highly Specific Escherichia Coli QPCR and Its Comparison with Existing Methods for Environmental Waters. *Water Res* **2017**, *126*, 101–110. https://doi.org/10.1016/j.watres.2017.08.032.

(21) Rose, J. B.; Haas, C. N.; Regli, S. Risk Assessment and Control of Waterborne Giardiasis. *Am J Public Health* **1991**, *81* (6), 709–713. https://doi.org/10.2105/AJPH.81.6.709.

(22) Center for Advancing Microbial Risk Assessment (CAMRA). Escherichia coli: Dose Response Experiments http://qmrawiki.org/experiments/escherichia-coli.

(23) TEUNIS, P. F. M.; OGDEN, I. D.; STRACHAN, N. J. C. Hierarchical Dose Response of E. Coli O157:H7 from Human Outbreaks Incorporating Heterogeneity in Exposure. *Epidemiol Infect* **2008**, *136* (6), 761–770. https://doi.org/10.1017/S0950268807008771.

(24) TEUNIS, P.; SCHIJVEN, J.; RUTJES, S. A Generalized Dose-Response Relationship for Adenovirus Infection and Illness by Exposure Pathway. *Epidemiol Infect* **2016**, *144* (16), 3461–3473. https://doi.org/10.1017/S0950268816001862.

(25) Navarro, I.; Jiménez, B.; Cifuentes, E.; Lucario, S. A Quantitative Microbial Risk Assessment of Helminth Ova in Reusing Sludge for Agricultural Production in Developing Countries. *Risk Anal* **2008**, *6*, 65–74.
